# High Rate of Recent Transposable Element–Induced Adaptation in Drosophila melanogaster

**DOI:** 10.1371/journal.pbio.0060251

**Published:** 2008-10-21

**Authors:** Josefa González, Kapa Lenkov, Mikhail Lipatov, J. Michael Macpherson, Dmitri A Petrov

**Affiliations:** Department of Biology, Stanford University, Stanford, California, United States of America; Duke University, United States of America

## Abstract

Although transposable elements (TEs) are known to be potent sources of mutation, their contribution to the generation of recent adaptive changes has never been systematically assessed. In this work, we conduct a genome-wide screen for adaptive TE insertions in Drosophila melanogaster that have taken place during or after the spread of this species out of Africa. We determine population frequencies of 902 of the 1,572 TEs in Release 3 of the D. melanogaster genome and identify a set of 13 putatively adaptive TEs. These 13 TEs increased in population frequency sharply after the spread out of Africa. We argue that many of these TEs are in fact adaptive by demonstrating that the regions flanking five of these TEs display signatures of partial selective sweeps. Furthermore, we show that eight out of the 13 putatively adaptive elements show population frequency heterogeneity consistent with these elements playing a role in adaptation to temperate climates. We conclude that TEs have contributed considerably to recent adaptive evolution (one TE-induced adaptation every 200–1,250 y). The majority of these adaptive insertions are likely to be involved in regulatory changes. Our results also suggest that TE-induced adaptations arise more often from standing variants than from new mutations. Such a high rate of TE-induced adaptation is inconsistent with the number of fixed TEs in the D. melanogaster genome, and we discuss possible explanations for this discrepancy.

## Author Summary

Transposable elements (TEs) are present in virtually all species and often contribute a substantial fraction of the genome size. Understanding the functional roles, evolution, and population dynamics of TEs is essential to understanding genome evolution and function. Much of our knowledge about TE population dynamics and evolution comes from the studies of TEs in *Drosophila*. However, the adaptive importance of TEs in the *Drosophila* genome has never been assessed. In this work, we describe the first comprehensive genome-wide screen for recent adaptive TE insertions in D. melanogaster. Using several independent criteria, we identified a set of 13 adaptive TEs and estimate that 25–50 TEs have played adaptive roles since the migration of D. melanogaster out of Africa. We show that most of these adaptive TEs are likely to be involved in regulatory changes and appear to be involved in adaptation to the temperate climate. We argue that most identified adaptive TEs are destined to be lost from the D. melanogaster population but that they do contribute significantly to local adaptation in this species.

## Abstract 3

Transposable elements contributed substantially to the adaptation of*D. melanogaster* to the out-of-Africa environments. The majority of these adaptive insertions are likely to be involved in regulatory changes.

## Introduction

The recent years have seen a burst in studies searching for signatures of genetic adaptation in a variety of organisms, including natural populations and domesticated plants and animals [1–12]. These studies suggest that adaptation is a pervasive force in evolution. However, many fundamental questions, for instance, the relative contribution of coding versus regulatory changes, point mutations versus structural changes, or different functional genic classes to adaptation, remain largely unanswered. Despite its central significance for all of biology, the genetics of adaptation remains very poorly understood.

One question that is still unanswered is the role that transposable elements (TEs) play in adaptation. One might expect that TEs participate in adaptation since TEs are potent sources of mutation and are known to contribute to the function and evolution of genes and genomes in a variety of ways [13–15]. TEs (1) play an important role in the structural evolution of genomes through the generation of various types of rearrangements [14,16], (2) donate regulatory sequences that control the expression of nearby genes [17–20], (3) become incorporated into coding sequences at the transcript level [21–24], and (4) have their genes recruited by the host genomes for key functions [25].

A common genomic effect of TEs is the induction of mutations. For instance, in Drosophila melanogaster, TEs are responsible for approximately 80% of the visible spontaneous mutations [26–28]. Most of the TE insertions are found at low frequencies, suggesting that the majority of the mutations they generate are deleterious [29,30]. TEs may be deleterious because they disrupt genes, because the translation of TE-encoded proteins may be costly, and also because they may mediate deleterious chromosomal rearrangements [31]. Only a few examples of TEs found at high population frequencies have been reported [32–39]. In two cases, there is good evidence that these high-frequency TEs have been adaptive in the recent evolution of D. melanogaster [33,38]. However, a systematic search for adaptive TEs in the D. melanogaster genome has never been carried out.

D. melanogaster is a particularly good model to analyze the contribution of TEs to adaptive evolution since it has one of the highest-quality genome sequences and annotations of TEs in eukaryotes [40,41]. D. melanogaster is also a particularly good model to study specifically recent TE-induced adaptation, since this species, originally from sub-Saharan Africa, expanded its population size worldwide very recently [42,43]. It appears that the expansion out of Africa into Europe took place approximately 10,000–16,000 y ago or equivalently 0.1 to 0.3 *N*_e_ generations ago [44,45]. As a result, we might expect that adaptations to the out-of-Africa environments that D. melanogaster is likely to have experienced [1,4,46] might still be detectable as partial or complete selective sweeps [47]. In addition, it should be easier to carry out genetic, phenotypic, and functional analyses of recent TE-induced adaptations given that such TEs would still be segregating in the D. melanogaster population, allowing for straightforward genetic manipulations. Note that the inference of partial or complete selective sweeps is complicated by the bottleneck that D. melanogaster appears to have experienced during the spread out of Africa [44,45]. It has been shown that bottlenecks alone can produce patterns of nucleotide variability that mimic those expected under selection [4,46,48–50]. Demography must therefore be taken into account before making any inferences of selective sweeps due to putatively adaptive TEs.

We used the annotated TEs in Release 3 of the D. melanogaster genome [51] as the starting point for our search for TEs that contributed to recent adaptation outside of Africa. We provide evidence for a high rate of TE-induced recent adaptive changes. The analysis of the set of adaptive TE insertions allows us (1) to estimate the minimum contribution of TEs to adaptive evolution, (2) to gain insight into the type of genes that have been targets of positive selection, (3) to assess the relative contributions of adaptive evolution in coding versus regulatory regions, and (4) to estimate the relative importance of new mutations versus standing variation. The estimated rate of adaptive transposition is unexpectedly high and inconsistent with the relatively small number of fixed TEs in the D. melanogaster genome. We discuss the implications of these results for the understanding of adaptation in *D. melanogaster.*

## Results

### Data

The third release of the D. melanogaster genome sequence identified 1,572 TEs belonging to 96 distinct families scattered across the euchromatic portion of the genome [51]. These TEs were identified by using BLAST to compare a reference dataset of canonical TE sequences against the genomic sequence. Only the euchromatic TE sequences displaying over 90% identity over more than 50 base pairs of sequence with the canonical TEs have been included in this set [51].

**Figure 1:**
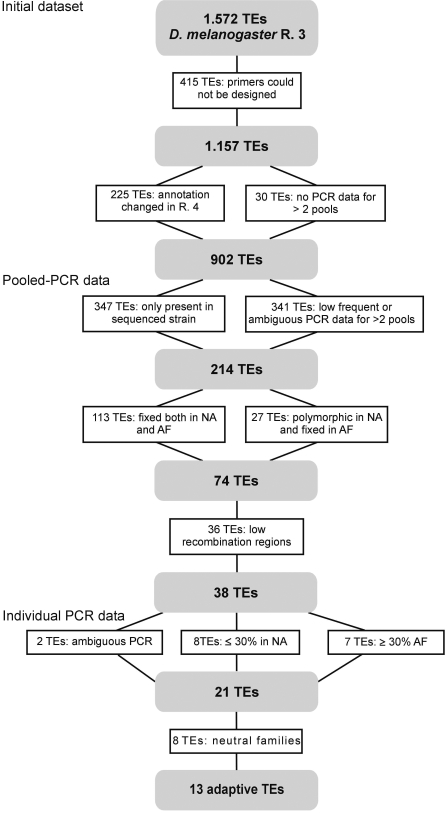
The Outline of the Procedure for the Identification of Putatively Adaptive Insertions

We used these 1,572 TEs as a starting point to determine population frequencies of the majority of euchromatic TEs. To obtain these frequencies, we employed a pooled-PCR strategy. PCRs were run with six DNA pools containing DNA from five different North American (NA) populations and one pool containing DNA from one sub-Saharan (Malawi) African (AF) population. Each DNA pool contained DNA from eight to 12 individual, isofemale, or highly inbred strains (see Materials and Methods). Not all the TEs in the Release 3 have been assayed—for 415 of them, specific primers could not be designed because the regions flanking the insertion were repetitive (see Materials and Methods). Release 4 of the D. melanogaster genome [41] corrected the annotation of approximately half of the elements in Release 3. The reannotation revealed that the primers had not been designed correctly for 225 TEs—we discarded the results for such TEs. As a result, we have information for 932 out of 1,572 TEs. For 695 of these TEs, we have information from all six NA pools, and for an additional 207 TEs, we have information from at least four NA pools. These 902 TEs form the starting point for our search for recent adaptive TE insertions (Figure 1; Table S1).

### Identifying TEs Frequent in NA by Pooled-PCR

Our goal is to identify TEs that may have contributed to adaptation after the expansion of the D. melanogaster population out of Africa. Therefore, we focused on identifying TEs that are rare or absent in Africa and are frequent or fixed in North America.

We start by identifying TEs present in all of the NA pools and not fixed in the AF pool. Specifically, we searched for insertions that (1) were clearly present in at least four NA pools, (2) were not clearly absent in any of the NA pools, and (3) were not fixed in the AF pool. Most of the 902 TEs are present at low population frequencies: 347 are only present in the sequenced strain, and another 341 TEs are either present at low frequencies in the five analyzed NA populations or gave ambiguous PCR results for more than two pools. A total of 214 TEs fulfill the first two criteria and therefore are more likely to be present at intermediate frequencies in the NA populations (Figure 1; Table S1); 113 of these 214 TEs appear fixed in all of the pools, including the AF pool. An additional 27 are polymorphic in NA pools but are fixed in the AF pool. Some of these TEs may have contributed to adaptation but are less likely to be recent and to have specifically contributed to adaptation associated with the out-of-Africa expansion. We eliminated these TEs from further consideration, leaving us with the set of 74 TEs.

Some of these remaining 74 TEs are present in the regions of low recombination (Table S1). TEs in the low-recombination areas are likely to be subject to weaker purifying selection due to a lower rate of ectopic recombination [35,52–54] and higher population stochasticity due to stronger background selection and stronger effect of linked positive selection [55–58]. The high-frequency TEs found in low-recombination areas are more likely to be neutral, and therefore to represent false positives, than those found in high-recombination areas. We eliminated the TEs present in low-recombination regions of the genome (<1.4 cM/Mb) from further analysis. At the end, based on pool frequency data, we identified 38 TEs that are located in regions of high recombination, are not fixed in sub-Saharan Africa, and which might be frequent in NA (Figure 1; Table S1). We focus on these 38 TEs for the remainder of this paper.

### Age of the TEs

We assessed the age of the 38 TEs by comparing their sequences to the consensus sequences of their families. We considered a TE insertion to be old when its divergence from the consensus sequence was higher than 1%. Using this criterion, we identified elements FBti0019418 and FBti0019634 from the *1360* family, FBti0019372 and FBti0020119 from the *S-element* family, and FBti0020114 and FBti0019081 from the *transib2* family as potentially old (Table 1).

**Table 1:**
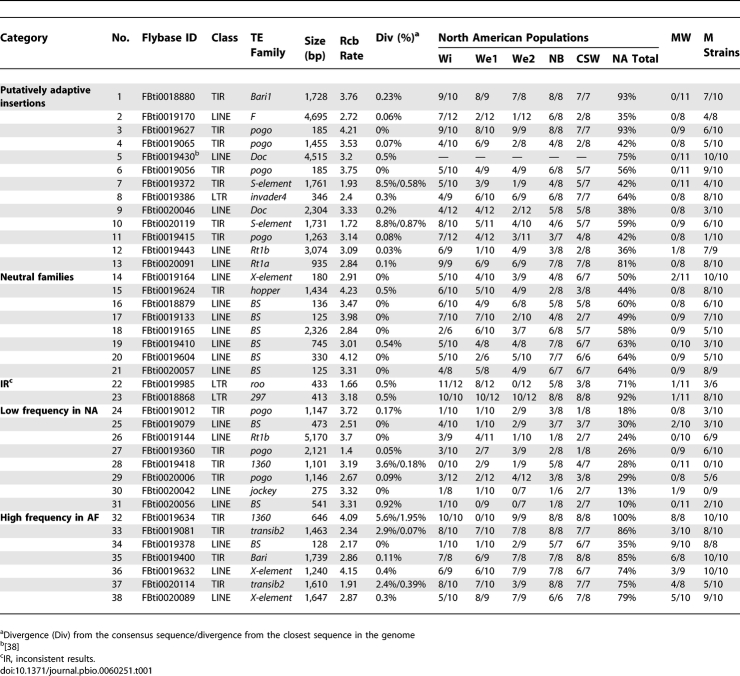
Individual Strain Frequency Data for the 38 TEs That Are Located in Regions of High Recombination and Are Likely to Be Frequent in NA and Rare in Sub-Saharan AF According to the Pooled-PCR Results

However, the possibility remains that these insertions are recent insertions of TEs whose sequence differ from that of the consensus. For the six putatively old insertions, we compared the sequence to other annotated TEs in the same family and also performed BLAST queries against the whole genome to search for closely related, but not annotated, copies (see Materials and Methods).

For FBti0019372, FBti0020119, FBti0020114, and FBti0019081, we found other elements in their respective families that showed less than 1% divergence, indicating that they are likely to be recent insertions (Table 1). FBti0019418 showed more than 1% divergence when compared to all the other identified copies belonging to the *1360* family. However, we discovered a new *1360* TE copy that is nested inside an element annotated as a *Cr1a* TE (FBti0059655) and is very similar to FBti0019418 (0.18% divergence). Only one *1360* copy (FBti0019634) is more than 1% divergent both from the *1360* consensus sequence and from any other *1360* copy in the genome. Consistently with its age estimate, FBti0019634 appears fixed in the AF pool (Table 1).

### Filtering out Rare TEs Using PCR with Individual Strains

The presence of a TE in all six NA pools suggests, but does not guarantee, that it is present at high frequency in the NA population. Indeed, a TE present at a 10% frequency in the population has an approximately 13% chance of being present in all six pools containing 12 strains each. To filter out the TEs present at a low frequency in the NA population and to verify the pooled-PCR results, we carried out PCRs with individual strains for all 38 putatively frequent TEs.

Results are shown in Table 1. Overall, we confirmed the results obtained with the pooled-PCR strategy. For two elements, FBti0020042 and FBti0020056, we could not detect the presence of the TE in any of the tested strains within a pool. In both cases, we can explain this by the inability to test every strain from the original pools because some strains were no longer available.

For most of the PCRs, we obtained a single band of the expected size, indicating that the primers were specifically amplifying the region of the genome where the TE was identified. We only found three exceptions. For a *pogo* element, FBti0019627, we obtained several bands besides the expected band for the presence of the element in all the strains assayed. We cloned and sequenced these amplification products and identified the band that contained this particular TE. We considered FBti0019627 to be present only when the PCR amplification products contained this specific band (Table 1).

For a *297* element, FBti0018868, and for a *roo* element, FBti0019985, the results obtained with the primers designed to check for the presence of the TE were not consistent with the results obtained with the primers designed to check for its absence. FBti0019985 also showed variability in the amplicon length. The specific reasons for these results are being currently investigated. These two elements were not considered further in this analysis (Table 1).

Of the remaining TEs, a number are rare in North America and/or frequent in Africa. We used an ad hoc cutoff of 30% to define frequent TEs. Using this cutoff, we eliminated eight TEs present at 30% or lower frequency in the NA strains and seven TEs present at 30% or higher frequency in the AF strains (Table 1). At the end, we have 21 TEs for which we have unambiguous evidence that they are frequent in North America and rare in Africa.

### Selection Coefficients of the TE Families

Some TEs belong to families in which the majority of copies are present at high frequency in the NA population and thus are unlikely to be adaptive. Instead, it is more plausible that such TE families are subject to relaxed purifying selection as a whole [35]. Using a maximum likelihood approach (see Materials and Methods), we estimated the selection coefficient for the 11 families represented in our list of 21 putatively adaptive TEs based on the NA pooled PCR data (Table 1). Three of the families, *BS*, *X-element*, and *hopper*, show selection coefficients that are not significantly different from zero, indicating that these families are likely to be under relaxed purifying selection (Table S2). For one of these families, the *BS* family, we have additional sequencing data that show that a number of the TEs in this family appear to have increased in frequency neutrally [50]. Eight elements in our list belong to one of these three families (Table 1). We considered these eight TEs to be putatively neutral and the remaining 13 TEs to be putatively adaptive (Figure 1).

### Presence of the Putatively Adaptive TEs in African Populations

Only one of the 13 putatively adaptive insertions is present in the analyzed AF population. However, we sampled only 11 Malawi strains. In addition, there might be substantial structure in the D. melanogaster population in sub-Saharan Africa [59] that might be further exacerbated by natural selection acting on the putatively functional TE insertions studied here. Moreover, we already know that one TE in our set, FBti0019430, is absent in the analyzed Malawi population but is present in 17% of the strains from a population collected in Kenya [38]. For the 11 TEs that were not present in the Malawi population, we extended the analysis to three other sub-Saharan populations: two from Zimbabwe and one from Kenya (Table 2). Seven out of 11 insertions were present in at least one of the pools assayed. Only two of them, FBti0018880 and FBti0019372, are absent in all three additional pools of AF strains. No results were obtained for the remaining two TEs (Table 2). We conclude that most putatively adaptive TEs are present in sub-Saharan Africa.

**Table 2:**
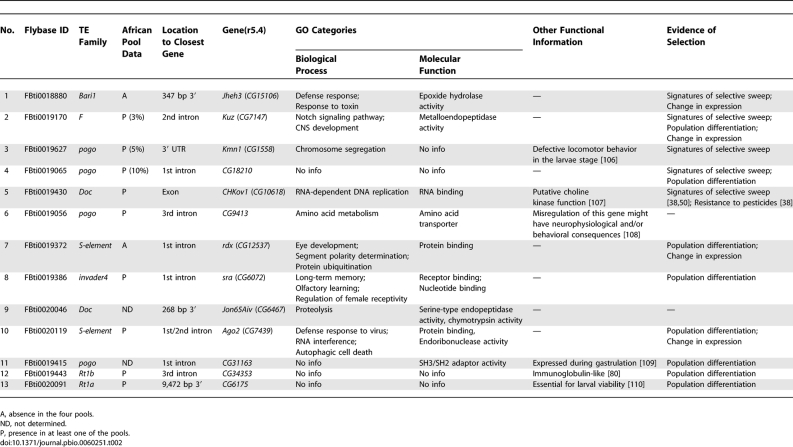
The 13 Adaptive TE Insertions Identified in This Work

### Signatures of Positive Selection in the Regions Flanking the Putatively Adaptive TEs

We investigated whether the identified 13 TEs are truly adaptive by searching for signatures of a partial selective sweep in the regions flanking the TEs. We sequenced the flanking regions in four out of the 13 insertions. Two of these four TEs, a *Bari1* element (FBti0018880) and a *pogo* element (FBti0019627) (Table 1), are present in 93% of the assayed NA strains. We also sequenced two TEs present at lower frequencies: another *pogo* element (FBti0019065) and an *F* element (FBti0019170). These insertions were found in 42% and 35% of the assayed NA strains, respectively (Table 1).

First, we performed individual strain PCRs for the three additional AF populations to estimate the frequency of these four TEs in the AF populations. FBti0018880 is absent in all the tested strains, and the three other insertions, FBti0019065, FBti0019170, and FBti0019627, are present at low frequencies (3% to 10%) in the tested AF populations (Table 2). Therefore, we are confident that they have increased in frequency either during or after the expansion out of Africa.

**Figure 2:**
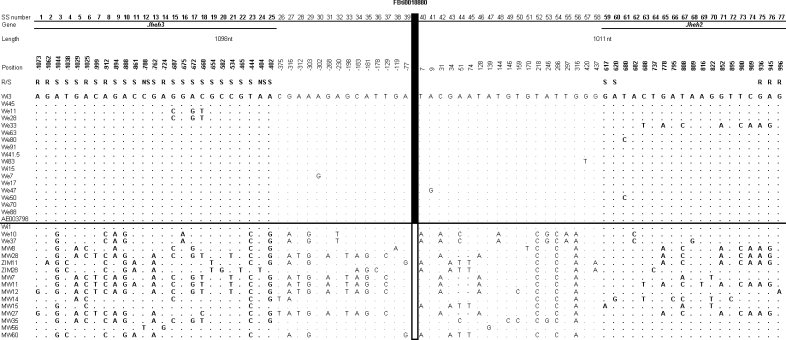
Sequence of the 2.1-kb Region Flanking FBti0018880 The figure shows the segregating sites (SS) within the 2.1-kb region flanking this insertion. The SS number, the gene associated with the insertion, and the length of the sequenced region are at the top. The distance from FBti0018880 is also shown. The SS within coding regions are in bold and are identified as replacement (R), synonymous (S), or nonsense (NS) polymorphisms. The TE is shown as a black rectangle; the absence of the TE is shown as an empty rectangle. A horizontal line separates the strains with the insertion from the strains without the insertion.

Figures 2 through 5 show the sequencing data for the flanking regions around these four TEs. The sequences from the strains with and without the TEs are separated by a black line, and the filled-in box indicates the position where the TE is inserted. A summary of the sequencing data is given in Table 3. FBti0019627 was present in all but three of the assayed NA strains (Table 1). We sequenced the three strains that did not contain the element: Wi98, We4, and We47. In two cases, We4 and We47, we found evidence for an independent excision event of the TE. Both strains contain the two-nucleotide target site duplication (TA) and two nucleotides that belong to the TE. We only consider a strain not to have the insertion if it does not show any evidence of excision. Therefore, we excluded these two sequences from the subsequent analysis.

**Figure 3:**
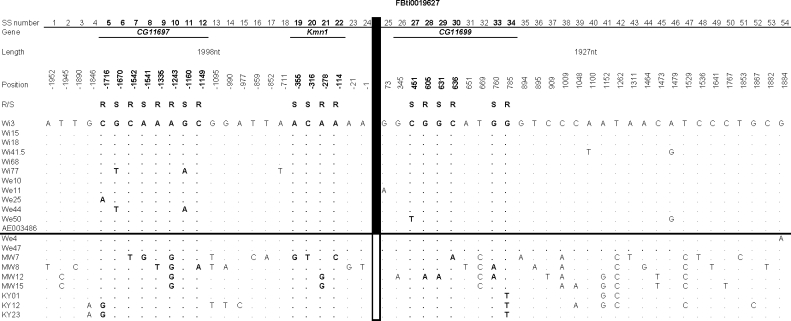
Sequence of the 3.9-kb Region Flanking FBti0019627. See Figure 2 for details.

**Figure 4:**
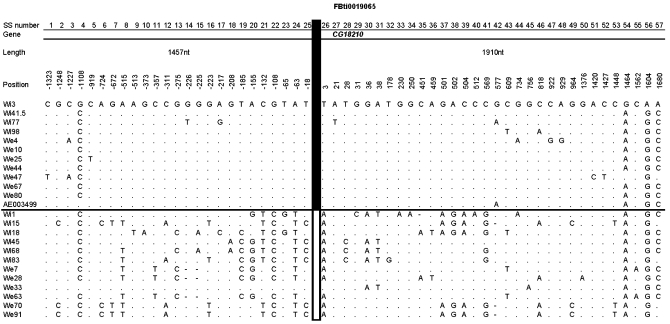
Sequence of the 3.4-kb Region Flanking FBti0019065. See Figure 2 for details.

**Table 3:**
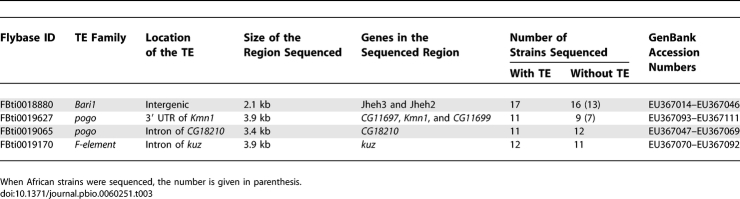
Summary of the Sequencing Data

As can be seen in Figures 2 through 5, similar polymorphism patterns are found around all four analyzed TEs. The strains with the TE show a reduced amount of polymorphism and fewer haplotypes compared to the strains without the insertion. These observations are consistent with the expectations of a selective sweep. However, the recent bottleneck likely experienced by the NA strains [42–45] can produce patterns on DNA sequence variation that mimic signatures of positive selection in a population of constant size [8,48–50,60,61]. Specifically, a search for the D. melanogaster TEs (or any polymorphisms) that are rare in the ancestral AF population and are common in the derived NA populations is expected to bias the results toward finding patterns resembling those of partial selective sweeps [50].

Macpherson et al. [50] employed coalescent simulations to explore how ascertainment biases, demography, purifying selection against the TE, and suppression of recombination caused by the TE affect the interpretation of polymorphism data. They analyzed the flanking sequences of five TEs. One TE belongs to our set of 13 putatively adaptive TEs—it is a *Doc* element (FBti0019430) that is quite likely to be adaptive as it disrupts a conserved gene and is linked to resistance to organophosphate and carbamate pesticides [38] (Y. T. Aminetzach, T. Karasov, and D. A. Petrov, unpublished data). The other four are *BS* elements: FBti0018879, FBti0019133, FBti0019410, and FBti0019604. These four BS elements belong to our set of eight putatively neutral TEs (Table 1). Macpherson et al [50] showed that the null model of neutrality and constant population size was strongly rejected for all five datasets. However, when the null models included the demographic scenarios specified in Thornton and Andolfatto [45] or Li and Stephan [44], only the presumably adaptive TE insertion (FBti0019430 [38]) showed signatures of positive selection. Incorporating purifying selection and recombination suppression to the null model strengthens this result although it did not change the conclusions qualitatively [50].

In view of these results, we decided to explore whether the haplotype configuration of the four insertions sequenced in this work depart from neutrality by considering a null model that incorporates the bottleneck scenario specified in Thornton and Andolfatto [45] and ascertainment of a derived polymorphism at a prespecified frequency matching that found in the data (see Materials and Methods). We estimated several statistical measures of polymorphism and compared them with the distributions obtained by simulation under this null model (Table 4 and Table S3). The integrated haplotype score (iHS) statistic is expected to be the most powerful indicator of a partial selective sweep [8]. We also estimated the proportion of nucleotide diversity within the haplotypes linked to the TE relative to the total nucleotide diversity in the sample, *f*_TE_ = π_TE_/(π_TE_ + π_non−TE_). Table 4 shows iHS and *f*_TE_ statistics both for the four elements sequenced in this work and for the five other elements studied previously [50]. In all five putatively adaptive cases, we found significant departures from neutrality. The *f*_TE_ values observed for the elements FBti0019065 and FBti0019170 are seven and four standard deviations away from the expected values, respectively. The iHS statistic for these two TEs was not significant potentially because only NA strains were sequenced [62]. For the other two TEs, FBti0018880 and FBti0019627, the iHS statistic showed significant deviations in the direction expected under a partial selective sweep. The observed values were five and four deviations away from the expectation, respectively. These results demonstrate that all five investigated putatively adaptive TEs show stronger signatures of positive selection than four investigated putatively neutral TEs. This suggests that the rest of the 13 insertions might be highly enriched for adaptive TEs as well.

**Table 4:**
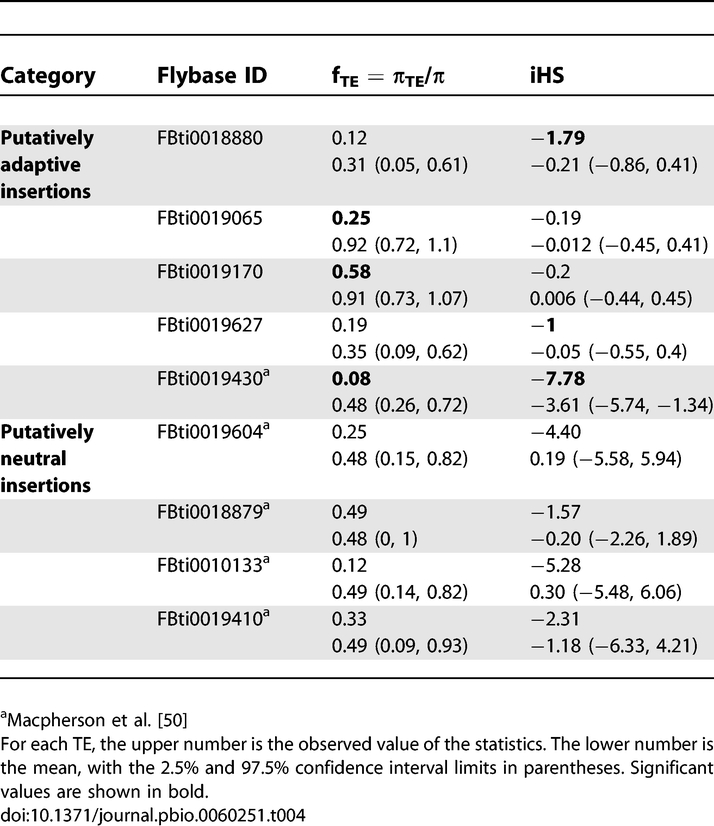
Neutrality Tests for Each of the Four TEs Sequenced in This Work

The sequencing allowed us to determine whether the four newly sequenced TEs were the causative agents of the sweeps rather than being passively linked to such causative mutations. In all four cases, the TE was located in the center of the apparent sweep, with the haplotype structure decaying on both sides. It is theoretically possible that the TE is in perfect linkage with a causative polymorphism located in the immediate vicinity of each TE. However, we did not find any such polymorphism in any of the four datasets.

### Estimating the Age of the Sweeps

The age of a partial selective sweep can be estimated by measuring the extent to which linkage disequilibrium decays at a known distance from the presumed focal site of adaptation [63]. For all four studied TEs, we sequenced 500-bp regions at approximately 10 kb away from each TE in several strains with and without the insertion (see Materials and Methods). We used the method of Slatkin and Rannala [63] to estimate that the partial selective sweeps associated with the elements FBti0019065 and FBti0019627 are approximately 0 to 500 y old, and those associated with the elements FBti0018880 and FBti0019170 are approximately 0 to 800 y old. Although being rough estimates of the age of the alleles, they agree with the scenario in which these partial selective sweeps have taken place after the out-of-Africa expansion of D. melanogaster.

Testing for the presence or absence of these TEs in the M strains can also yield insight about the time of the spread of the TEs in the NA population. M strains are old laboratory stocks that were established before the 1940s and can be molecularly defined by the absence of the *P* elements in their genome [64]. Therefore, TEs found at a high frequency in the modern D. melanogaster populations, but which are absent or rare in the M strains, most likely have increased in frequency in the last 70 y.

We checked the frequency of the 13 putatively adaptive TEs in ten M strains originally sampled from around the world (see Materials and Methods). All 13 TEs are present in the M strains at frequencies comparable to those found in the recently sampled NA strains (Table 1). Thus, there is no evidence of very recent expansions of these TEs. We have also investigated the M strain frequency for all 38 TEs present in the initial list of putatively adaptive TEs (Table 1). Only two TEs, FBti0020042 and FBti0019418, are absent in all of the M strains assayed (Table 1). However, these two TEs are present at low frequencies in the modern NA strains, suggesting that all the TEs in the list of possibly adaptive TEs have reached their current frequencies prior to the 1940′s.

### Evidence of Population Differentiation for the 13 Putatively Adaptive Elements

The results of the haplotype tests described above are suggestive of positive selection. However, they should not be taken as conclusive evidence for selection since the true demographic model for D. melanogaster is unknown. Moreover, the frequency of the TE in the ancestral population and the extent of recombination suppression in heterozygotes due to the presence of the TE are also unknown. We know that the variation of these parameters might affect the distribution of tested statistics under neutrality and thus affect our inference of positive selection [50]. Consequently, we decided to perform an additional, independent test of the adaptive role of these elements—whether the frequencies of these TEs are higher in more temperate compared to more tropical out-of-Africa populations of D. melanogaster. Such a pattern would be expected if these TEs provide adaptive benefits in the temperate, but not in the tropical, habitats.

We analyzed the frequency of the 21 TEs, including the 13 putatively adaptive and the eight putatively neutral TEs, in 44 strains from two Eastern Australian populations. These two populations are located close to the ends of a latitudinal cline along the Eastern coast of Australia [65]. The Northern population is located near Innisfail, Queensland, where the climate is similar to that of the likely ancestral, sub-Saharan African habitat. The Southern population is located at the Yering Station, Victoria, and has a much colder, less tropical climate characteristic of the more temperate out-of-Africa–derived habitats. Note that D. melanogaster likely colonized Australia only 100 y ago, most likely through a single northern invasion [65], and that the Australian population had not been used by us for the identification of the 13 putatively adaptive TEs. Thus, the differentiation of the TE frequencies across these two populations would serve as an independent test of adaptation both in the historical and the experimental sense.

We used a maximum likelihood procedure to estimate the frequencies of the TEs in the two Australian populations (see Materials and Methods). The set of 13 adaptive TEs shows significant heterogeneity of frequencies between these two populations (*p* < 0.0001), whereas the set of eight putatively neutral TEs does not show such heterogeneity (*p* = 0.19). Moreover, only one of the eight putatively neutral elements showed heterogeneity in its population frequency (*p* = 0.009), whereas eight out of the 13 elements in the putatively adaptive set showed such heterogeneity (*p* < 0.05) (Figure 6; Table S4). We tested whether there is significantly more differentiation for the putatively adaptive TEs compared to the putatively neutral TEs, and we found that indeed this is the case (*p* = 0.023, *G*-test with Yates correction for continuity). In all nine instances of significant heterogeneity, the TE frequency was higher in the temperate Southern population compared to the tropical Northern population, consistent with our expectations.

Three of the eight putatively adaptive TEs and the one putatively neutral TE that showed population differentiation are located inside the cosmopolitan chromosomal inversion *In(3L)Payne* or *In(3R)Payne* (Table S4). Both inversions show latitudinal patterns in Australian populations (see Hoffmann and Weeks for a review [65]). Only one of these two inversions, *In(3L)Payne*, has been characterized at the molecular level and therefore can be scored by PCR (see Materials and Methods). We checked for the presence of this inversion in the 44 strains analyzed in this work and found that it is only present in one strain. It so happens that the two putatively adaptive TEs that are located inside this inversion (FBti0020091 and FBti0020119) failed to be amplified in this particular strain. Therefore, we can conclude that the presence of *In(3L)Payne* is not affecting our results. For the other two TEs that showed population differentiation and are included in *In(3R)Payne*, we cannot discard the potential confounding effects of *In(3R)Payne* on their population frequency. The exclusion of these two TEs does not affect the significance of the comparison of the putatively adaptive and putatively neutral TEs, however. There is still significantly more differentiation for the putatively adaptive TEs (*p* = 0.014, *G*-test with Yates correction for continuity). Note also that the TEs showing heterogeneity are distributed across all three major chromosomes and are unlinked with each other, suggesting that these patterns are independent cases of adaptive differentiation between these two populations (Table S4).

### Analysis of the Putatively Adaptive Elements

The 13 TEs included in our list of putatively adaptive insertions belong to eight different families from all three major classes of TEs: long terminal repeat (LTR) retrotransposable elements (one family), long interspersed nucleotide element (LINE)-like retroposons (four families), and DNA transposons with terminal inverted repeats (TIR) (three families) (Table 1). Some numerous families such as *roo* or *jockey* are not represented, whereas other families like *pogo*, *Doc*, or *S-element* contribute more than one element [51] (Table 1). LTR elements, the most abundant class of elements in the genome [51], are significantly underrepresented in our set (*p* = 0.006). The 13 putatively adaptive TEs are evenly distributed among the chromosomal arms (*p* = 0.57).

We wanted to test whether the 13 putatively adaptive TEs (the “adaptive” set) are peculiar in any way compared to the putatively nonadaptive TEs (the “nonadaptive” set) within the same families that are also found in regions of high recombination (95 TEs total). Specifically, we focused on three properties: size, distance to the closest flanking gene, and functional properties of the flanking genes.

We compared the size of the TEs in the adaptive and nonadaptive sets to the canonical size of their families [51]. We classified the elements into three categories: near full length (>90% of the canonical length), medium length (20%–90% of canonical length), and small (<20% of the canonical length) (Table S1). TEs in the adaptive set are not significantly different in size from the TEs in the nonadaptive set (χ^2^ = 0.5; *p* = 0.778).

Most of the known *cis*-regulatory sequences in *Drosophila* are located within 1 kb of the transcriptional start site [66]. Taking this into account, we classified the TEs into three categories in relation to their distance to the nearest gene: inside genes, located less than 1 kb from a gene, and located more than 1 kb from a gene (Table S1). Again, we failed to detect any differences in the distribution of these distances for the TEs in the adaptive and nonadaptive sets (χ^2^ = 0.75; *p* = 0.687).

Finally, we analyzed the functional association of the genes found next to the TEs in our two sets using the Gene Ontology (GO) database. Some of the genes next to the adaptive TEs are associated with more than one GO term, a representative list of which is given in Table 2. Ten of the 13 genes have a GO term for the biological process and/or the molecular function categories. For example, three of them are associated with genes involved in response to stimulus: FBti0018880, FBti0019386, and FBti0020119 (Table 2). We used FatiGO+ [67] to search for terms that are significantly over- or underrepresented in the adaptive set compare to the nonadaptive set. The biological process term “response to stimulus” appears overrepresented in the set of genes associated with the putatively adaptive TEs (*p* = 0.003). However, the false discovery rate–adjusted *p*-value is above 0.05. In conclusion, none of the terms in the molecular function or cellular component categories is over- or underrepresented in the adaptive set. It is possible that the failure of FatiGO+ to find significant differences between the two sets of genes is at least partly due to the small size of the adaptive TE set further compounded by the sparse functional and molecular annotations of the neighboring genes in both sets.

Finally, for the 13 putatively adaptive insertions, we searched for expressed sequence tags (ESTs) containing both the TE and the gene they are associated with in the Ensembl database [68]. Only insertions FBti0019430 and FBti0019627 form chimeric transcripts with genes *CHKov1* and *Kmn1*, respectively, supported by EST evidence. For the rest of the TEs, we did detect ESTs that contain part of or the whole TE sequence, but none of these ESTs also contain genic sequence. We found no ESTs for FBti0018880.

### Expression Analysis of the Genes Located Closest to the Putatively Adaptive TEs

Twelve out of 13 adaptive TEs are located outside of coding regions, in many cases in close proximity to genes or inside introns. This suggests that the adaptive effects of these TEs are likely due to their effects on gene regulation. To confirm this inference, we analyzed the expression of the 12 genes located closest to the putatively adaptive TEs (Table 2). The 13th TE (FBti0019430) is inserted into the exon of *CHKov1* and had been previously shown to truncate the original *CHKov1* protein and to generate a new functional protein [38]. For each tested gene, we searched for differences in expression between the allele carrying the TE and the allele lacking the TE in the F_1_ heterozygous adults. Differential expression of the two alleles in the same cellular environment of the F_1_ individual is indicative of functional *cis*-regulatory differences [69].

For each TE, we identified two highly inbred NA strains that both differ by the presence/absence of this TE and by the presence/absence of a diagnostic SNP in the coding region of the adjacent gene. The exact procedure is described in Materials and Methods. Pyrosequencing was then used to measure the relative abundance of the two alleles [69]. This technique has been demonstrated to be a sensitive tool to quantify allele-specific expression, enabling discrimination of subtle differences in transcript abundance [70]. We obtained data for five genes (Figure 7; Table S5). Four of them showed differences in expression between the two alleles: in three cases, the allele of the nearby gene that carries the TE in *cis* is down-regulated, and in one case, it is up-regulated. As can be seen in Figure 7, for some genes, the results depend on the direction of the cross, suggesting that there could be a parental effect on the regulation of the genes close to the TE. These results further suggest that the majority of the recently adaptive TEs in D. melanogaster have an effect on the expression of the adjacent genes.

## Discussion

### Screening for Recent Adaptive Insertions in the *Drosophila* Genome

The spread of D. melanogaster out of sub-Saharan Africa within the last 10 to 20 thousand years ago exposed D. melanogaster to new ecological and physiological challenges. These new challenges likely led to adaptive genetic changes in the non-African populations of D. melanogaster. In this study, we set out to search for such recent adaptations driven specifically by insertions of TEs.

We started our search from a set of 1,572 individual insertions annotated in Release 3 of the D. melanogaster genome [51]. Starting from a set of TEs found in the sequenced genome biases our ascertainment toward preferentially finding TEs present at higher population frequencies. All things being equal, we are bound to find every fixed TE; on average, 50% of TEs present at 50% frequency in the population; 10% of TEs at 10% frequency; and so on. However, in addition, the choice of the sequenced strain introduces its own bias. The sequenced strain of D. melanogaster (*y^1^; cn^1^ bw^1^ sp^1^*) is an old laboratory strain likely to have been isolated from the wild at the beginning of the 20th century in the United States [71]. Therefore, our ascertainment is biased toward finding TEs that were frequent in NA populations at the beginning of the century. These biases are in many ways helpful for the search of the TEs that contributed to the out-of-Africa adaptation of D. melanogaster. Indeed, such TEs should be frequent in the NA populations and would be discovered at a reasonable chance using our procedure. We would miss all of the very recent adaptations, however.

We obtained population frequency data for 902 TEs both in NA and AF populations and found that most of these 902 TEs are present at low population frequencies. These results confirm previous findings based on the analysis of individual families suggesting that in *Drosophila*, TEs are under purifying selection [29,30]. Based exclusively on their population frequencies, we identified 13 TEs in the highly recombining regions of the D. melanogaster genome that could plausibly play a role in the out-of-Africa adaptation (Table 2). Note that we define high-recombination areas as those where recombination rate is greater than 1.4 cM/Mbp. However, our results are not very sensitive to the exact value of the cutoff. Varying the cutoff between 1 and 2 cM/Mbp changes the number of putatively adaptive TEs from 13 to 11. These 13 TEs are segregating at high frequencies (>30%) in North America and at low frequencies (<30%) in Malawi. They also belong to the TE families that appear to be evolving under purifying selection in general, making it less likely that these 13 TEs rose to high frequencies by genetic drift alone. We also identified eight TEs that are also frequent in North America and rare in Africa but which belong to TE families that are under reduced purifying selection. This makes these eight TEs more likely to be neutral. We use them as a control set of putatively neutral TEs in our population genetic analyses.

**Figure 5:**
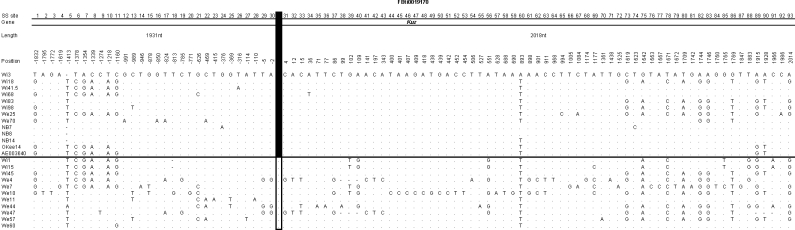
Sequence of the 3.9-kb Region Flanking FBti0019170. See Figure 2 for details

To start testing whether at least some of these 13 TEs are in fact adaptive, we sequenced the flanking regions of four of them (Figures 2–5; Table 2). We used coalescent simulations to test whether the nucleotide polymorphism pattern surrounding these four TE insertions depart significantly from a null model that incorporates the demographic scenario specified in Thornton and Andolfatto [45] and ascertainment of a derived polymorphism at a prespecified frequency matching that found in the data [50]. Another putatively adaptive TE (FBti0019430) had been sequenced previously along with four out of the eight neutral TEs we have identified here [50]. We did find significant departures from neutrality in the direction expected under a partial selective sweep for all five tested putatively adaptive TEs, but not for four putatively neutral TEs (Table 4). This provides highly suggestive evidence for the role of positive selection in the increase of frequency of the majority of the putatively adaptive TEs. Different statistics were significant for the different TEs: *f*_TE_ was significant for FBti0019065 and FBti0019170, and iHS was significant for FBti0018880 and FBti0019627. Both *f*_TE_ and *iHS* are significant for FBti0019430. The observation that not all of the tests are significant for each TE is not entirely unexpected given that these TEs were already present in Africa prior to the putative partial sweeps. Simulations have previously demonstrated that positive selection from standing variation may not leave as strong a signature in the patterns of linked polymorphisms as positive selection acting on de novo mutations [62,72,73].

**Figure 6:**
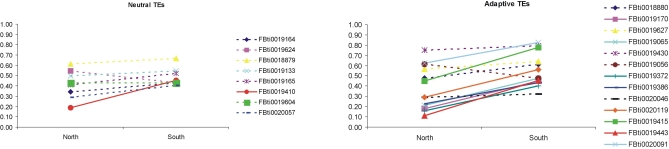
Population Frequency of the Eight Putatively Neutral and 13 Putatively Adaptive TEs in Two Australian Populations Collected Close to the Extremes of a Latitudinal Cline The North population was collected in Innisfail (Queensland) and the South population in Yering Station (Victoria).

**Figure 7:**
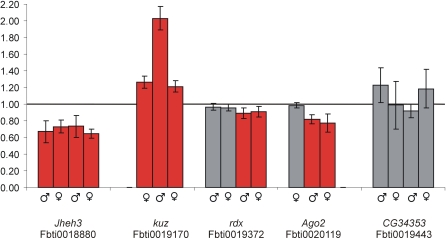
Normalized Allelic Ratios for the Five Genes Analyzed in This Study For each gene, the first two bars correspond to the F_1_ progeny (male and female, respectively) of the cross in which the parental female is homozygous for the presence of the TE. The last two bars correspond to the progeny of the cross in which the parental male is homozygous for the presence of the TE. For genes *kuz* and *Ago2*, the PCR with one of the four samples failed. Significant ratios are represented by red bars and nonsignificant ones by grey bars.

We used the sequencing data to also assess whether these TEs are likely to be causative adaptive mutations or whether they just happen to hitchhike to high frequencies with linked adaptive mutations. In all five studied cases, (1) the TE appears to be completely linked to the partial sweep, (2) the partial sweep decays on both sides of the TE, and (3) there are no polymorphism other than the TE itself that are in perfect linkage disequilibrium with the partial sweep. These results suggest strongly that the TE is likely to be the causative mutation of the partial sweep rather than to be merely linked to such a causative mutation.

The signatures of selective sweeps in the regions flanking the putatively adaptive TEs provide some evidence for their adaptive increase in frequency but should be treated with caution. The uncertainty about the starting frequency of the TE in the ancestral population and about the appropriate demographic model makes it difficult, if not impossible, to come with very robust neutral expectations about the distributions of tested statistics [50]. We therefore performed an additional independent test of the adaptive role of these elements.

These 13 TEs are expected to be adaptive in the environments characteristic of the out-of-Africa expansion, but not adaptive in Africa. We thus expect these TEs to be less frequent in the out-of-Africa populations located in the more tropical regions compared to the populations located in the more temperate regions. To test this prediction, we analyzed the frequencies of the 13 putatively adaptive and eight putatively neutral TEs in two populations located close to the ends of a latitudinal cline in the Eastern coast of Australia. The Northern populations experience tropical climates, whereas the Southern ones experience more temperate ones. Consistent with our predictions, eight of 13 putatively adaptive TEs are significantly more frequent in the Southern population, whereas only one of eight neutral ones shows such differentiation (Figure 6; Table S4). We also ensured that these patterns are not due to the linkage of these TEs to inversions that show clinal patterns of variation along the Eastern coast of Australia [65] or to each other. Note that because D. melanogaster colonized Australia less than 100 years ago and because we did not use the Australian population data in defining the set of adaptive and neutral TEs, these results provide a powerful independent test of adaptive significance of the 13 identified TEs.

The evidence of the partial selective sweeps due to all five putatively adaptive TEs tested combined with the population heterogeneity between tropical and temperate habitats for eight out of 13 putatively adaptive TEs indicate very strongly that most, if not all, of the identified 13 TEs play adaptive roles in the out-of-Africa D. melanogaster population.

### Nature of TE-Induced Adaptations: Regulatory Changes Are Predominant

The analysis of the location of the 13 recent adaptive insertions identified in this work gives insight into the relative contribution of protein-coding versus regulatory changes in adaptation [74,75]. Most of the TEs in our set are located in introns or intergenic regions (eight and three TEs, respectively), whereas only two are located in the mature transcripts: one within an exon (FBti0019430) and one in a 3′ UTR (FBti0019627; Table 2). This distribution suggests that recent adaptive insertions are mostly involved in regulatory changes.

To further explore this possibility, we analyzed the expression of the genes located next to the adaptive insertions. Changes in gene expression can arise from *cis*-regulatory changes that affect transcription and/or transcript stability in an allele-specific manner, or from *trans*-regulatory changes that influence expression of both alleles [69,76]. We searched for *cis*-regulatory differences by comparing the relative abundance of transcripts in F_1_ hybrids in which one allele contains the TE in *cis* and the other one does not. In four out of the five genes for which we obtained results, we showed that the expression of the allele carrying the TE in *cis* is significantly different from the expression of the allele lacking the TE (Figure 7). In three cases, the expression was down-regulated, and in one case, it was up-regulated. These results support the role of the adaptive TEs in the regulation of the adjacent genes and agree with the analysis of chimeric gene–TE proteins in the human genome, suggesting that the role of young TEs is probably most often limited to regulatory functions [23].

### Nature of the Genes and Pathways Associated with the Adaptive TE Insertions

The analysis of the types of genes associated with the 13 adaptive insertions might provide an insight into the type of biological processes that have been targets of selection in the expansion of D. melanogaster out of Africa (Table 2). Three of the genes associated with our set of adaptive insertions are involved in processes grouped under the GO category “response to stimulus”: *Ago2*, *sra*, and *Jheh3* (Table 2). This category has been previously associated with genes under positive selection in *Drosophila* [33,38,77,78]. Another three genes, *kuz*, *rdx*, and *Jon65Aiv*, are associated with protein metabolism. An overrepresentation of genes associated with protein metabolism has been found in the analysis of genes likely to be under positive selection after the expansion of D. simulans out of Africa [79]. However, there is no overlap between the two gene datasets, suggesting that the same type of biological processes, but not exactly the same genes, have been the target of selection in the expansion of both D. melanogaster and D. simulans out of Africa.

For some of the genes that do not have a GO annotation, there is additional information that suggests the biological processes in which they might be involved. For example, *CG34353* has been described as an immunoglobulin-like (Ig-like) gene [80]. Many of the proteins in the Ig-like family are cell surface or secreted proteins that have important roles during development. Such genes have been previously shown to exhibit signatures of positive selection [81,82].

Some of the adaptive TEs are located close to or inside genes belonging to highly conserved pathways. Such insertions are likely to be involved in the fine-tuning of these processes. For example, FBti0018880 is inserted in the 0.7-kb intergenic region between *Jheh2* and *Jheh3* genes and down-regulates at least one of them (*Jheh3*, Figure 7). Both of these genes are involved in Juvenile Hormone (JH) metabolism [83]. This hormone has major effects on various aspects of development and life history, not only in *Drosophila*, but also in other insects [84]. FBti0019170 is inserted in the intron of *kuz*, a gene in the *Notch* (*N*) signaling pathway, and up-regulates it (Figure 7). *N* is a transmembrane receptor that mediates local cell–cell communication and coordinates a signaling cascade present in all animal species studied to date [85]. Finally, FBti0019372 is inserted in the first intron of *rdx*, a gene involved in the *Hedgehog* (*Hh*) signal transduction pathway [86] and down-regulates it. *Hh* plays essential roles in a multitude of developmental processes via a complex signaling cascade conserved from insects to mammals [87].

Overall, there is no clear overriding pattern in the types of genes that are located near the adaptive TEs. It is possible that the number of adaptive TEs is too small or our understanding of the functional role of many genes is too limited to see this pattern. Future investigation of the functional effects of the adaptive TEs will be required to understand the phenotypic and ecological nature of adaptation due to these TEs.

### Origin of TE-Induced Adaptations: Local Adaptation from Standing Variation

Adaptive mutations can arise in two different ways. On the one hand, adaptation can start out as a new mutation that is favored as soon as it arises. Most of the searches for recent adaptations are guided by this model of positive selection [88–90]. However, this assumption may not be realistic, especially if adaptation takes place in response to range expansions. Environmental changes associated with range expansions can lead to previously neutral or slightly deleterious alleles that were segregating in the ancestral population to become beneficial [48,72,73]. This seems to be the scenario for the majority of the 13 adaptive TEs described here. We found that the majority of them were already present in the ancestral AF populations (Table 2). Only two out of 13 putatively adaptive TEs were absent from all four sub-Saharan AF populations, suggesting that the majority of recent TE-induced adaptations in D. melanogaster came from standing variation.

Furthermore, all 13 adaptive TEs are very similar in their sequence from the other TEs in their families (divergence less than 1%), suggesting that these 13 TEs inserted into the genome very recently (Table 1) and therefore are unlikely to have been subject to long-term balancing selection. Hence, it appears that these TEs were either neutral or slightly deleterious in the ancestral African population and became adaptive upon the expansion of D. melanogaster into temperate habitats.

### Rate of TE-Induced Adaptive Evolution

The goal of this study was to identify recent TE insertions highly likely to be adaptive in the recent evolutionary past of D. melanogaster. We followed a conservative approach that undoubtedly led us to miss some adaptive insertions. For example, since we focused on recent insertions likely to have contributed to adaptation during or after the expansion of D. melanogaster out of Africa, we ignored all TEs present at high frequencies in the Malawi population. These insertions are less likely to have increased in frequency specifically in the out-of-Africa populations. However, some of these TEs may still have contributed to adaptation in the out-of-Africa populations. For example, all parallel TE-induced adaptations in the African and out-of-Africa populations will be missed by this approach. We will also miss all of the TEs that contributed to adaptation prior to the expansion of D. melanogaster out of Africa.

There are 114 insertions that appear fixed in all of the analyzed populations; 25 of them are located in regions of high recombination and therefore are more likely to be enriched for adaptive insertions (Table S1). We also did not consider insertions present at high frequencies in genomic regions characterized by low recombination rates since they are more likely than those found in high-recombination areas to be neutral [55–58,91]. However, some of these insertions, particularly the ones that are present at high frequencies in the NA populations and absent in the AF populations, are also likely to be adaptive. There are 15 such insertions; nine of them are most probably not adaptive since they belong to families under relaxed purifying selection (D. A. Petrov, J. González, M. Lipatov, A. S. Fiston-Lavier, and K. Lenkov, unpublished data), but the other six TEs might be adaptive (Table S1). All of these TEs deserve further study.

As stated before, the starting point of our search for adaptive TEs were the insertions described in one D. melanogaster strain that was probably collected at the beginning of the last century in North America. This ascertainment bias implies that we are undercounting some, especially less frequent TEs. Given the frequency distribution of the 13 putatively adaptive TEs and the PCR failure rate, we can estimate that the NA populations at the beginning of the century had approximately 25 adaptive TEs in the high-recombination regions of the genome. If we suppose that the rate of adaptation is the same in low- and high-recombination regions, then as many as 50 TE insertions anywhere in euchromatin have been adaptive since the out-of-Africa migration of D. melanogaster. Note also that we are missing all TEs that may have contributed to adaptation in other out-of-Africa populations but were rare in NA at the beginning of the century. For instance, the TEs that increased in frequency after the collection of the sequenced strain are bound to be missed by this study. As a case in point, we did not sample the insertion of an *Accord* TE previously found to confer resistance to insecticides because it is not present in the sequenced genome [33] and is rare in the M strains in general (Y. T. Aminetzach, T. Karasov, and D. A. Petrov, unpublished data).

Thus, the list of 13 putatively adaptive TEs is likely an underestimate of all adaptive TEs. We can consider that at least 13, and more likely 25–50, adaptive TEs have increased in frequency in the NA populations since the expansion of *Drosophila* out of Africa approximately 10,000 to 16,000 y ago [44,45] and before the collection of the sequenced strain (∼70 y ago). This corresponds to one adaptive TE increasing to intermediate frequencies in the D. melanogaster euchromatin every 200 to 1,250 y.

If all of the identified TEs are destined to reach fixation and the rate of adaptation was similarly high prior to the expansion of D. melanogaster out of Africa, then this rate appears incompatible with the number of fixed TEs in the D. melanogaster genome. Indeed, even if we conservatively estimate that we should only be able to detect TEs fixed within the past approximately 1 million years (Myr) (corresponding to the expected neutral divergence of ∼3%), we should see 800 to 2,500 fixed TEs in euchromatic regions of high recombination and up to 5,000 TEs in euchromatin in general. This assumption is conservative, given that all TEs less than 10% divergent from its consensus sequence are expected to be found and the average time to loss of 50% of the DNA in *Drosophila* is substantially greater than 1 Myr [92]. In contrast to this large expected number of fixed TEs, only 25 fixed insertions in high-recombination regions of the genome, and 114 in total, have been detected.

There are at least three distinct, but not mutually exclusive, scenarios that would explain why we see so few fixed TEs in the D. melanogaster genome. First, it is possible that the rate of adaptation is not constant. The rate that we estimated could be reflecting a burst in adaptations that took place during the expansion of D. melanogaster out of Africa. A higher rate of adaptive evolution in the derived populations compared to the African populations could be expected and in fact has been suggested by previous studies [44].

Second, it is likely that these TEs are adaptive in some, but not other, environments. Supporting this, we found that eight of them appear to be adaptive to temperate climates (Figure 6). Moreover, we did not find any TE fixed in the NA populations of D. melanogaster and polymorphic or absent in AF. Our estimates of the frequencies of these 13 TEs in the M strains also show that the current frequencies have been stable for the last 70 y (∼700 generations) (Table 1). If this is the explanation for the observed low number of fixed TEs, then our results suggest that the majority of local adaptations are destined to be lost. Such local adaptations might be common for other, non–TE-derived recent adaptations and, similar to the TE-derived adaptations, they might be ephemeral.

Finally, we might be underestimating the number of fixed insertions in the genome if the adaptive TEs undergo faster sequence divergence compared to the neutral TEs. This is not entirely far-fetched as newly adaptive TEs might undergo a bout of fast sequence changes driven by positive selection. If many of these adaptive substitutions are indels, then the sequence of the TEs might quickly become obscured. A more sensitive search for degenerate TE sequences in the D. melanogaster genome might be productive in this case.

### High Rate of Adaptation in *Drosophila*

Our estimate of the rate of TE-induced adaptations, one every 200–1,250 y or one every 2,000–25,000 generations, suggests that, at least since the expansion of D. melanogaster out of Africa, TEs have contributed considerably to adaptive evolution. Several recent studies based on the analysis of both coding [2,3,9] and noncoding regions [5] suggest that the genomic rate of adaptive evolution is high. For example, Smith and Eyre-Walker [2] estimated that approximately 45% of amino acid substitutions in *Drosophila* were driven by positive selection, which translates into one adaptive substitution every 450–900 generations. This rate is even higher, approximately one adaptation every 70–520 generations when only the noncoding regions of the genome are considered [5]. The above estimates focus on adaptations that fix in the genome. Using a different approach, based on the spatial correspondence between neutral polymorphism and nonsynonymous divergence, Macpherson et al [12] also argued for a high rate of adaptive substitution. These authors estimated that approximately one adaptation every 3,000 generations is taking place in the *Drosophila* species. The rate of TE-induced adaptation is of the same order of magnitude and thus might be a significant source of adaptive mutations in *Drosophila*.

The high rate of adaptation estimated in these various studies is surprising. In order to increase our confidence in these estimates and to understand the nature of adaptation, it is clearly important to connect putatively adaptive mutations to their phenotypic effects. The adaptive TE insertions that we identified in this study represent a promising set for such functional analyses. Again, most of the adaptive insertions identified in this paper are closely linked to genes of at least partly known functional roles. For example, insertion FBti0018880 is likely to affect the expression of genes involved in the degradation of JH. JH affects a significant number of processes and traits in *Drosophila* development and life history, including metamorphosis, behavior, reproduction, diapause, stress resistance, and aging [84]. Any of these processes could have been affected by the insertion of this TE in this particular region of the genome. They are therefore likely candidates to be analyzed in order to assess the functional consequences of the insertion. For the insertions closely located to genes with no functional information, components of fitness such as male and female fertility, survival rates through development or temperature and desiccation resistance can be studied.

### Conclusions

A systematic identification of adaptive insertions described in this work allows us to infer that TEs are a considerable source of recently adaptive mutations in the *Drosophila* genome. Most of the adaptive TEs are located close, but not inside, protein coding regions of genes and appear to affect the expression of these genes. Functionally diverse genes located next to the putatively adaptive TEs provide a rich collection for a follow-up investigation of adaptive processes in D. melanogaster. The adaptive TE insertions appear to have been present in Africa as neutral or deleterious polymorphisms prior to the expansion of D. melanogaster out of Africa and are only adaptive in some, specifically temperate environments. The high rate of recent adaptive changes due to TEs appears to be incompatible with a low number of fixed TEs in the D. melanogaster euchromatin. This most likely indicates that most locally adaptive TEs are destined to be lost over long periods of time. It is tempting to speculate that such local adaptations (1) are common for other types of mutations as well and (2) tend to be ephemeral and lost fairly quickly in general. This would imply that genetic variation within species might often be due to different mutations than that between species. Thus, the extent to which functional genetic variation within species is ephemeral rather than “a phase in molecular evolution” [93] remains to be determined.

## Materials and Methods

### Pooled-PCR frequency data.

DNA from five different NA populations (8–12 strains per population; 64 strains in total) and one AF population collected in Malawi (11 strains) were combined into seven different pools. Six pools contained DNA from the NA populations, and one pool contained DNA from the AF population. The composition and the geographical origin of each pool is given in Table S6. Strains from the Wi pool were subject to over 30 generations of brother–sister matings. Strains from the We1 and We2 pools were subject to 10–15 generations of brother–sister matings. Strains from NA, NB, and CSW pools are isofemale strains. The final concentration of DNA in each pool was 2.5 ng of each individual strain per PCR reaction. Genomic DNA from all these strains was extracted using DNeasy Tissue kit (Qiagen).

The absent/polymorphic/fixed status of each TE in all the pools was determined using the polymerase chain reaction (PCR). All PCR primers were designed using Primer 3 [94] and were checked with Virtual PCR [95]. One set of primers was intended to assay for the presence of the TE insertion and consists of a “Left” (L) primer which lay within the TE sequence and a “Right” (R) primer that lay in the flanking region to the right of the TE insertion. We expect this PCR to give a band only when the element is present. The other set of primers was intended to assay for the absence of the TE insertion and consisted of a “Flank” (FL) primer which lay in the left flanking region of the TE sequence and the R primer mentioned above. In this case, the absence of a TE in the pool should give a shorter, “absence” band, and the presence of a TE should give a longer, “presence” band. We assumed that the presence band is unlikely to be amplified if the TE sequence is longer than 800 bp. For the insertions that overlap with another TE, specific R or FL primers could often not be designed, and therefore, the frequency of such TEs was not assayed.

PCR reaction mix was made using Redtaq Readymix from Sigma Aldrich and primers at a final concentration of 1 μM/μl. The PCR conditions were: 94 °C for 5 s, 27 cycles of 94 °C for 30 s, 62 °C for 30 s, and 72 °C for 1 min. We classified an element as absent when the L-R primer pair did not yield a band, and the FL-R primer pair yielded an absence band only. We classified an element as polymorphic if the combined L-R and FL-R primer pairs produced both a presence and an absence band. Finally, we classified an element as fixed if the L-R primers yielded a presence band and the FL-R primers yielded either a presence band or no band at all (if the element is longer than 800 bp, the FL-R primers were not expected to amplify the presence band). For the TEs shorter than 800 bp, the failure of FL-R primer was interpreted as PCR failure and the PCR results as ambiguous. Here, we only analyzed in detail those TEs for which both primer pairs gave a mutually consistent result.

### Individual strain frequency data.

The same two sets of primers described above were used to detect both the presence and the absence of a subset of the TEs in each individual strain present in the different pools (Table S6).

Besides the above strains, for some of the insertions, three additional AF pools were assayed. Two of the pools contained strains collected in Zimbabwe, and the other one contained strains collected in Kenya. The composition of these three pools is also given in Table S6.

In addition, we used ten M strains from Bloomington *Drosophila* Stock Center at Indiana University that were collected worldwide: Canton-S, Oregon-R-C, Oregon-R-S, Amherst 3, Lausanne-S, Samarkand, Swedish-C, ORiso-2, CSiso-2, and Berlin-K. First, we confirmed that these were truly M strains by checking for the presence/ absence of *P* elements. As a positive control, we used a classic P strain (Harwich stock, also from Bloomington *Drosophila* Stock Center at Indiana University). An inverted repeat–specific primer of the D. melanogaster canonical *P* element was used [96]. Amplification consisted of a first step of 7 min at 94 °C and then 30 cycles of 45 s at 94 °C, 45 s at 57 °C, and 1.5 min at 72 °C. A final extension step at 72 °C for 10 min was carried out.

Finally, we also checked the frequency of a subset of the TEs in two Australian populations collected in 2007 close to the ends of a latitudinal cline: Innisfail in far North Queensland, and Yering Station in South Victoria. For each population, a total of 22 stocks were analyzed (Table S6). For these 44 stocks, we also checked for the presence of inversion *In(3L)Payne*. Primers were designed in the region spanning the distal breakpoint of this inversion [97]. Primer pair 5′-CCGGATGGACCACATAGAAC-3′ and 5′-CATTCTGGGCCTTATCATCT-3′ amplify the standard, but not the inverted, chromosome. Primer pair 5′-CCGCAAACGAACACTTA-3′ and 5′-GATTATGGACCTAATGAAAGC-3′ amplify the inverted, but not the standard, chromosome.

For all the individual strain PCRs, the following conditions were used: 94 °C for 2 min, 13 cycles of 94 °C for 30 s, 63 °C for 30 s (−0.5 °C per cycle), 72 °C for 1 min, and then 20 cycles of 94 °C for 30 s, 56 °C for 45 s, 72 °C for 1 min, and one last extension step of 10 min at 72 °C.

### Dating the insertions.

For each of the elements for which we obtained individual strain frequency data, levels of divergence from their consensus sequence (available at http://flybase.bio.indiana.edu) were estimated. Sequences were aligned using Sequencher software (v. 4.7; Gene Codes Corporation). The minimum size of the aligned regions was 180 bp. We considered a TE to be old if its divergence from the consensus sequence is greater than 1%. However, it could also be that these apparently old insertions are recent insertions generated by active TEs whose sequence differs from the consensus sequence of the family. To test for this possibility, we aligned the insertions showing greater than 1% divergence from the consensus sequence to the rest of sequences that belong to the same families. To detect the existence of copies closely related to our insertions that have not been previously identified, we performed BLAST queries against the whole D. melanogaster genome with the sequence of these apparently old insertions. We calculated pairwise distance using Mega 3.1 [98] and identified the sequence most closely related to our insertion. We then estimated the percentage of divergence between those two sequences.

### Maximum likelihood estimation of selection coefficients.

For each group of TEs, we performed nested likelihood analysis, following the work of Petrov et al. [35]. Assuming that all TEs within a family are subject to uniform selection with a selection coefficient *s* along with a heterozygous effect *h* = 1/2, and given the size of the D. melanogaster population *N*, we can calculate the sojourn time of each new insertion at any given frequency *x*—i.e., the time this insertion is expected to spend in a short interval between *x* and *x* + Δ*x*. To do so, we made use of a diffusion approximation and the resulting sojourn time density function (equations 4.22 and 4.23 in Ewens [99]) [35]:


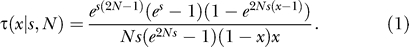


The probability that a randomly chosen TE insertion will be found at frequency *x* is proportional to the above sojourn time *τ*(*x*). However, the insertions we studied were all originally found in the sequenced strain. Thus,

Pr[an insertion we detect is at frequency *x*] = Pr[we detect the insertion | the insertion is at frequency *x*] × Pr[a random insertion is at frequency *x*] ∝ *x* × *τ*(*x | s*, *N*) ≡ *α*(*x | s*, *N*).

In the above, we defined α(*x* | *s, N*), a function that is proportional to the probability that any given insertion is at frequency *x*. The probability itself, then, is this function normalized by its integral over all possible values of *x*:


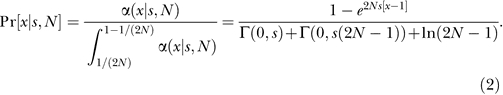


Here, we integrated *α*(*x* | *s, N*) between 1/(2N) and 1 − 1/(2N), because in reality, a polymorphic element insertion cannot be present at frequencies that are outside this range. The incomplete gamma function that appears in the denominator is given by Γ(*a*,*x*) ≡ 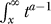 *e* ^−*t*^ *dt*.

For each TE, our data come in the form *m* ≡ {*m*_1_, *m*_2_, *m*_3_, *m*_4_, *m*_5_, *m*_6_}, where *m*_1_ is the number of NA strain pools in which the element is absent, *m*_2_ is the number of pools in which it is polymorphic, and *m*_3_, the number of pools in which it is fixed. *m*_4_ and *m*_5_ give the numbers of pools with partial information—those where the element is either absent or polymorphic, and those where the element is either polymorphic or fixed. Finally, *m*_6_ is the number of pools about which we have no reliable information. The sum of *m*_1_ through *m*_6_ is always equal to 6, since that is the number of NA strain pools.

Note that the numbers of strains vary between eight and 12 for different pools. However, the estimates of selection coefficients and population frequencies consistently vary by a factor of less than 1.5 as we switch between these two pool sizes. Consequently, in the following treatment, we adopted an intermediate value of 11 strains per pool, close to the pool average.

In the following, we also need to consider the fact that some of the pools in which the element is polymorphic, may appear to have the element either as absent or fixed. We estimate that the rates of both types of errors are approximately equal to 0.046 (unpublished data).

Conditional on *x*, the element's frequency in the population, for the pools in which we can distinguish perfectly between the three classifications (absent, polymorphic, and fixed), the probability of *m*_1_ pools classified as absent, *m*_2_ as polymorphic, and *m*_3_ as fixed is


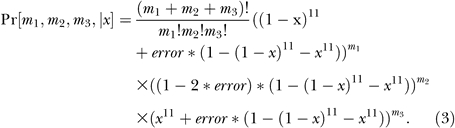


The probability of finding that the element is absent or polymorphic (as opposed to fixed) in *m*_4_ pools is


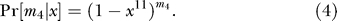


Finally, the probability of finding that an element is polymorphic or fixed (as opposed to absent) in *m*_5_ pools is


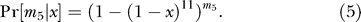


Combining Equations 2 through 5, and integrating over the entire range of possible element frequencies in the population, we get the total probability of obtaining the data (*m*_1_ through *m*_5_), given a selection coefficient *s* and population size *N*.


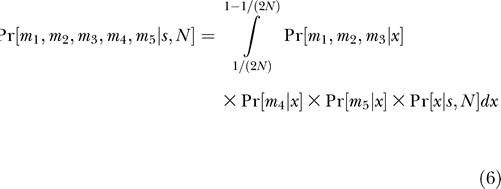


Since we assumed that the same selection coefficient and population size apply to every element in the group, the combined probability of obtaining a particular set of data for all the group's elements is


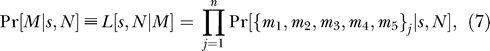


where *M* denotes the combined set of data for the *n* elements in a group, and {*m*_1_, *m*_2_, *m*_3_, *m*_4_, *m*_5_}*_j_* is the data for a given element *j*. We noted that this is also the likelihood of the population genetic parameters *s* and *N* given the data, i.e., L[*s*,*N* | *M*].

Previous studies showed that the size of the NA D. melanogaster population is likely to be between 10^5^ and 10^6^ [100,101]. Furthermore, we have shown that the qualitative conclusions of our analysis do not change as we switch between these two values [35]. Accordingly, we proceeded by fixing *N* at 10^5^ in the probability distributions and likelihood functions below. For each of the family/recombination rate groups, we found the value of *s* that gives us the maximum likelihood of the group's combined data (Equation [7]).

We then constructed a likelihood model in which each element within a group may come from one of two subgroups with different selection coefficients *s*_1_ and *s*_2_. The probability that each element comes from a subgroup with a selection coefficient *s*_1_ is *p*, and that it comes from the other subgroup is 1 − *p*. In order to get the probability of element's frequency *x* under the new model with three parameters, instead of one, we extended Equation 2 as follows:


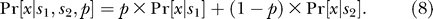


Using Equation 8, we constructed the new likelihood function analogous to the one described by Equations 6 and 7:


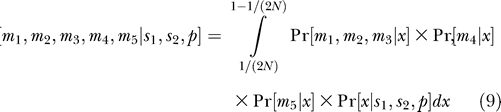


and


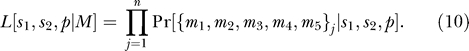


For each group of TEs, we found parameters *s*_1_, *s*_2_, and *p* that maximize this new likelihood function, and wanted to test whether the improvement in likelihood is significant from the value we obtained for a one-parameter model. To do this, we used the likelihood ratio test, which involves calculating


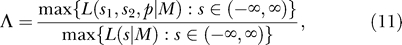


and comparing −2*ln(Λ) to the χ^2^ distribution with the number of degrees of freedom equal to the difference in the numbers of parameters between the two models. In practical terms, since that difference is 2, and the 95th percentile of the corresponding distribution is equal to 5.991, we need ln(Λ) to increase additively by at least 5.991/2 = 2.996 as we increase the number of parameters in our model. Whenever we saw such an improvement, we interpreted it as evidence of heterogeneity of selection coefficients within a group of TEs.

We went on to construct a likelihood model with five parameters: *s*_1_, *s*_2_, *s*_3_, *p*_1_, and *p*_2_. Here, *s*_1_, *s*_2_, and *s*_3_ are the selection coefficients of possible element subgroups, and *p*_1_ and *p*_2_ are the proportions of elements that come from the first two subgroups. However, the maximum likelihood value under this model never showed improvement above the threshold value of 2.996 (see above) for any of the TE groups we considered.

Finally, we estimated the confidence intervals on the selection coefficients. In case of either of the two likelihood functions above (either with one or with two selection coefficients), we calculated the confidence intervals on each selection coefficient *s_i_* by holding all parameters except *s_i_* constant, and noting the values of *s_i_* where the function drops under two units below its maximum value. This procedure is based on a likelihood ratio test, where the test statistic is the likelihood ratio of the zero-parameter (or two-parameter) model with *s_i_* fixed at the value of its maximum likelihood estimate to the two-parameter (or three-parameter) model with *s_i_* that is allowed to vary. This statistic is distributed as a χ^2^ distribution with one degree of freedom. When the difference in log-likelihoods increases above two, the likelihood ratio increases above *e* ^2^ = 7.39, where *e* is the base of the natural logarithm. This value is the 99.3% quantile of the χ^2^ distribution (corresponding to *p* = 0.007, 1 *d.f.*).

### Sequencing the regions flanking the putatively adaptive insertions.

Based on the sequenced genome of D. melanogaster, we designed primers in an overlapping fashion to amplify the 5′ and 3′ flanking regions of four of the insertions: FBti0018880, FBti0019065, FBti0019170, and FBti0019627 (Table S7). For a particular insertion, the same set of primers were used to sequence both strains with and without the element except for the primer pairs amplifying the regions immediately 5′ and 3′ to the insertion: primer pairs FL and FL_R, and L and R were used only in the strains with the insertion, given that primers FL_R and L are designed inside the element. On the other hand, the primer pair FL and R was used to sequence the strains without the element.

Only populations from Davis, CA, and Raleigh, NC, appeared truly isogenic based on previous sequencing data [38]. For the rest of the strains, DNA was amplified using a proofreading DNA polymerase (Platinum Pfx; Invitrogen) and cloned into Zero Blunt TOPO PCR cloning kit (Invitrogen) before sequencing. The number of strains sequenced for each element varies between 20 and 33. For two of the elements, FBti0019065 and FBti0019170, only NA strains were sequenced, and for the other two elements, FBti0018880 and FBti0019627, both NA and AF strains were sequenced.

### Coalescent simulations and statistical tests of neutrality.

To assess the statistical significance of the polymorphism patterns in the TE datasets, we compared several summary statistics calculated over the datasets to distributions of these statistics obtained by neutral coalescent simulations. The statistics we computed included π [102], iHS [8], and *f*_TE_. We evaluated π over various subsets of the sequences: NA sequences, AF sequences, TE-bearing sequences, and non–TE-bearing sequences. iHS was calculated only with respect to the presence of the TE and without respect to whether a TE-bearing strain was NA or AF.

The coalescent simulations attempt to account for both the demographic history of D. melanogaster and the sample configuration, as follows. The simulations assume a demographic model derived from Thornton and Andolfatto [45]. In this model, the modern NA population derives from a founder population of African origin. We assume this emigration event to have occurred 0.022 *N*_e_ generations before the present, where *N*_e_ is the effective population size of the modern AF population. From 0.022 *N*_e_ generations until 0.0042 *N*_e_ generations before the present, the NA population is assumed to have size 0.03 *N*_e_. From 0.0042 *N*_e_ generations ago until the present, the NA population is assumed to have effective population size *N*_e_. The transitions in NA population size at 0.022 *N*_e_ and 0.0042 *N*_e_ occur instantaneously. No migration occurs between the NA and AF populations from 0.022 *N*_e_ generations until the present. The African population size *N*_e_ is assumed to remain constant throughout. These estimates correspond to the high-recombination scenario (ρ = 10) considered in Thornton and Andolfatto [45].

We accounted for the sample configuration using a simple acceptance–rejection algorithm. We set the numbers of contemporary AF and NA strains to those obtained in TE-typing assays (Table 1). Many more strains were typed in these assays than were sequenced, which implies that the typing assays provide better estimates of the true TE frequency in each subpopulation, and this is why the typing assay values were used. Only simulations in which a derived-state allele could be found segregating in the exact numbers the TE was found to be segregating in the NA and AF population were accepted. Following this, the simulated sample was randomly pruned to match the sample configuration of the respective flanking-sequence dataset, such that the segregating site that matched the TE segregation pattern in the TE-typing assay now matched the TE segregation pattern in the respective flanking sequences. Finally, the sample was only accepted if it had the same number of segregating sites to the left and to the right, respectively, of the TE locus as in the actual sample. To improve the acceptance rate, we typically simulated conditional on some small multiple of the observed number of segregating sites, multiplying the length in base pairs of the sequence by the same value, and then truncating to the required number of segregating sites.

Coalescent simulations were conducted using the program ms [103]. In these simulations, *N*_e_ was set to 10^6^, and the respective estimates of local recombination rate reported in Table 1 were used. One thousand replicates were obtained for each locus.

### Estimating allele age.

We estimated the age of the TEs, i.e., the time elapsed since the TE inserted, based on the decay of linkage disequilibrium between the TE locus and “distal flanking sequences” of approximately 500 bp at loci roughly 10 kb away from the TE, using the method of Slatkin and Rannala [63]. Because this method requires that each distal sequence be classified as one of at most two alleles, we employed the resampling method of Tang et al. [104] to partition the sequences into two allelic groups. For both distal flanking sequence datasets, we performed for each of the four TE datasets, 1,000 replicates of the partition method, yielding a distribution of allele ages that accounts for the uncertainty in the partitioning. In the distal flanking alignments, any site having a gap was ignored. Primers used to amplify and sequence these regions are given in Table S7.

### Maximum likelihood estimation of TE population frequencies.

We estimated the frequency of each TE in the Australian populations and evaluated the heterogeneity of the frequencies between the Northern and the Southern populations using a maximum likelihood procedure. The Australian strains are not fully isogenized, as evidenced by the heterozygosity of many TEs for presence and absence in many strains (Table S4). We assumed that each tested strain effectively contains two different haploid genomes and that different strains within a tested set come from a panmictic population. The data for each TE in each population come in the form {*m*_1_, *m*_2_, *m*_3_}, where *m*_1_ is the number of strains homozygous for the presence of the TE, *m*_2_ is the number of strains heterozygous for the presence of the TE, and *m*_3_ is the number of strains that are homozygous for the absence of the TE. The log-likelihood of observing such data conditional on the frequency *p* is:


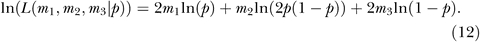


The *L*(*m*_1_,*m*_2_,*m*_3_ | *p* is maximized at the value *p̂*:


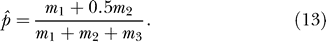


To determine whether the frequencies in the two tested populations are different from each other, we compare the log-likelihoods of two models. Under H1, we assumed that the frequencies in the two populations are different and estimated them using Equation 13 with the data that come from each population separately. We also calculated the two corresponding maximum log-likelihoods. Under H2, we assumed that the frequency of the TE is the same in both populations and estimate this frequency using Equation 13 with the combined data from the two populations. We also estimate the maximum log-likelihood under H2. The heterogeneity is detected when the difference between the sums of the two maximum log-likelihood values under H1 and the maximum log-likelihood value under H2 (denoted by Δ*L*) is greater than 3.84, corresponding to the 5% critical value of the χ^2^ test with one degree of freedom. The total heterogeneity across a group of TEs is evaluated by comparing the sum of the Δ*L* values for each element with the critical values of a one-tailed χ^2^ distribution with the number of degrees of freedom equal to the number of TEs in the group.

### Allele-specific expression analysis.

We analyzed the expression of the genes close to 12 of the 13 putatively adaptive TEs. FBti0019430 is inserted in an exon of gene *CHKov1* and has been shown previously to truncate this gene and generate a new functional protein [38]. Consequently, it was not included in this analysis.

First, we sequenced fragments of the coding regions of the genes next to the TEs, and we searched for SNPs in linkage disequilibrium with the TE. Primers used to amplify and sequence these genes are given in Table S8. For TEs FBti0018880 and FBti0019627, we used the SNPs previously discovered in this work (Figures 2 and 3). Sequencing these SNPs allowed us to distinguish between the mRNA originating from the chromosome carrying the insertion and the mRNA originating from the chromosome lacking the insertion.

Then, we established crosses between a strain homozygous for the presence and a strain homozygous for the absence of each adaptive insertion that also differ by the presence/absence of the diagnostic SNP. For each insertion, we established two different crosses: in one cross, the mother is homozygous for the presence of the element, and in the other cross, the father is homozygous for the presence of the element. The specific stocks used for each insertion are given in Table S9. We used the F_1_ progeny of these crosses to check for differences in expression between the allele carrying the insertion and the allele lacking the insertion. For each cross, we collected 3–5-day-old males and females and analyzed them separately. Flies were snap frozen in liquid nitrogen and stored at −80 °C until use. Total RNA was extracted using TRIzol reagent (Invitrogen). RNA was treated with DNase to remove any contaminating DNA and purified using RNeasy mini kit (Qiagen). The concentration of purified total RNA was determined spectophotometrically at 260 nm. First-strand cDNA synthesis was performed with SuperScriptIII First-Strand synthesis system for reverse transcriptase PCR (RT-PCR) (Invitrogen). To check for genomic contamination, RT-PCR controls without retrotranscriptase were performed. Specific primers to amplify and sequence each SNP were designed (Table S10). A universal sequence was appended to one primer of each set. PCR was done in the presence of 2.5 μM tailed primer, 10 μM nontailed primer, and 10 μM universal biotin labeled primer. Each sample was analyzed in triplicate.

Pyrosequencing of the PCR products was performed by EpigenDx. The use of this sequencing technique for gene expression analysis at the allele level has been shown to enable discrimination of subtle differences in transcript abundance [70]. The allelic ratio in the cDNA was normalized to remove systematic artifacts caused by unequal amplification or biases in peak heights due to inequality of light emission from incorporation of different nucleotides [105]. To do that, we used the same primers to amplify genomic DNA of the F_1_ adults. The allelic ratio in the cDNA was then normalized, taking into account the allelic ratio obtained for the genomic DNA. Significance was tested by an unpaired *t*-test since genomic DNA and cDNA come from different individuals.

## Supporting Information

Table S1 Data for the 902 TEs Analyzed in This Study (199 KB XLS)

Table S2 Estimates of the Selection Coefficients for the 11 Families Included in the Set of 21 Putatively Adaptive Elements (17 KB XLS)

Table S3 Additional Neutrality Tests for Each of the Four TEs Sequenced in This Work (29 KB DOC)

Table S4 Population Frequencies of the 13 Putatively Adaptive and Eight Putatively Neutral TEs in Two Australian Populations (23 KB XLS)

Table S5 Normalized Allelic Ratios for the Five Genes Analyzed in This Study (16 KB XLS)

Table S6 D. melanogaster Strains Used in This Study (36 KB DOC)

Table S7 Primer Pair Combinations Used to Sequence the Regions Immediately 5′ and 3′ to the Four Insertions Sequenced in This Work (66 KB DOC)

Table S8 Primer Pair Combinations Used to Discover SNPs in the Genes Located Close to Ten of the 13 Adaptive TEs (17 KB XLS)

Table S9 Stocks Used for the Crosses to Determine the Allele-Specific Expression of the 12 Adaptive TEs (15 KB XLS)

Table S10 Primer Pair Combinations Used to Amplify and Sequence SNPs in the Genes Located Close to the 12 Adaptive TEs (17 KB XLS)

## Abbreviations

AF: African
EST: expressed sequence tag
GO: Gene Ontology
iHS: integrated haplotype score
LINE: long interspersed nucleotide elements LTR, long terminal repeat
NA: North American
TE: transposable element
TIR: terminal inverted repeat

## References

[pbio-0060251-b001] Glinka S, Ometto L, Mousset S, Stephan W, De Lorenzo D (2003). Demography and natural selection have shaped genetic variation in Drosophila melanogaster: a multi-locus approach. Genetics.

[pbio-0060251-b002] Smith NG, Eyre-Walker A (2002). Adaptive protein evolution in Drosophila. Nature.

[pbio-0060251-b003] Bierne N, Eyre-Walker A (2004). The genomic rate of adaptive amino acid substitution in Drosophila. Mol Biol Evol.

[pbio-0060251-b004] Ometto L, Glinka S, De Lorenzo D, Stephan W (2005). Inferring the effects of demography and selection on Drosophila melanogaster populations from a chromosome-wide scan of DNA variation. Mol Biol Evol.

[pbio-0060251-b005] Andolfatto P (2005). Adaptive evolution of non-coding DNA in Drosophila. Nature.

[pbio-0060251-b006] Wright SI, Bi IV, Schroeder SG, Yamasaki M, Doebley JF (2005). The effects of artificial selection on the maize genome. Science.

[pbio-0060251-b007] Biswas S, Akey JM (2006). Genomic insights into positive selection. Trends Genet.

[pbio-0060251-b008] Voight BF, Kudaravalli S, Wen X, Pritchard JK (2006). A map of recent positive selection in the human genome. PLoS Biol.

[pbio-0060251-b009] Shapiro JA, Huang W, Zhang C, Hubisz MJ, Lu J (2007). Adaptive genic evolution in the Drosophila genomes. Proc Natl Acad Sci U S A.

[pbio-0060251-b010] Williamson SH, Hubisz MJ, Clark AG, Payseur BA, Bustamante CD (2007). Localizing recent adaptive evolution in the human genome. PLoS Genet.

[pbio-0060251-b011] Andolfatto P (2007). Hitchhiking effects of recurrent beneficial amino acid substitutions in the Drosophila melanogaster genome. Genome Res.

[pbio-0060251-b012] Macpherson JM, Sella G, Davis JC, Petrov DA (2007). Genomewide spatial correspondence between nonsynonymous divergence and neutral polymorphism reveals extensive adaptation in Drosophila. Genetics.

[pbio-0060251-b013] Kidwell MG, Lisch DR (2001). Perspective: transposable elements, parasitic DNA, and genome evolution. Evolution Int J Org Evolution.

[pbio-0060251-b014] Kazazian HH (2004). Mobile elements: drivers of genome evolution. Science.

[pbio-0060251-b015] Biemont C, Vieira C (2006). Genetics: junk DNA as an evolutionary force. Nature.

[pbio-0060251-b016] Hughes JF, Coffin JM (2001). Evidence for genomic rearrangements mediated by human endogenous retroviruses during primate evolution. Nat Genet.

[pbio-0060251-b017] van de Lagemaat LN, Landry JR, Mager DL, Medstrand P (2003). Transposable elements in mammals promote regulatory variation and diversification of genes with specialized functions. Trends Genet.

[pbio-0060251-b018] Jordan IK, Rogozin IB, Glazko GV, Koonin EV (2003). Origin of a substantial fraction of human regulatory sequences from transposable elements. Trends Genet.

[pbio-0060251-b019] Marino-Ramirez L, Lewis KC, Landsman D, Jordan IK (2005). Transposable elements donate lineage-specific regulatory sequences to host genomes. Cytogenet Genome Res.

[pbio-0060251-b020] Lowe CB, Bejerano G, Haussler D (2007). Thousands of human mobile element fragments undergo strong purifying selection near developmental genes. Proc Natl Acad Sci U S A.

[pbio-0060251-b021] Nekrutenko A, Li WH (2001). Transposable elements are found in a large number of human protein-coding genes. Trends Genet.

[pbio-0060251-b022] Lipatov M, Lenkov K, Petrov DA, Bergman CM (2005). Paucity of chimeric gene-transposable element transcripts in the Drosophila melanogaster genome. BMC Biol.

[pbio-0060251-b023] Gotea V, Makalowski W (2006). Do transposable elements really contribute to proteomes. Trends Genet.

[pbio-0060251-b024] Piriyapongsa J, Rutledge MT, Patel S, Borodovsky M, Jordan IK (2007). Evaluating the protein coding potential of exonized transposable element sequences. Biol Direct.

[pbio-0060251-b025] Volff JN (2006). Turning junk into gold: domestication of transposable elements and the creation of new genes in eukaryotes. Bioessays.

[pbio-0060251-b026] Green MM (1988). Mobile DNA elements and spontaneous gene mutation. Banbury Rep.

[pbio-0060251-b027] Sankaranarayanan K (1988). Mobile genetic elements, spontaneous mutations and the assessment of genetic radiation hazards in man. Banbury Rep.

[pbio-0060251-b028] Ashburner M, Golic KG, Hawley RS (2005). Drosophila: a laboratory handbook.

[pbio-0060251-b029] Charlesworth B, Langley CH (1989). The population genetics of Drosophila transposable elements. Annu Rev Genet.

[pbio-0060251-b030] Charlesworth B, Sniegowski P, Stephan W (1994). The evolutionary dynamics of repetitive DNA in eukaryotes. Nature.

[pbio-0060251-b031] Nuzhdin SV (1999). Sure facts, speculations, and open questions about the evolution of transposable element copy number. Genetica.

[pbio-0060251-b032] Maside X, Bartolome C, Charlesworth B (2002). S-element insertions are associated with the evolution of the Hsp70 genes in Drosophila melanogaster. Curr Biol.

[pbio-0060251-b033] Daborn PJ, Yen JL, Bogwitz MR, Le Goff G, Feil E (2002). A single p450 allele associated with insecticide resistance in Drosophila. Science.

[pbio-0060251-b034] McCollum AM, Ganko EW, Barrass PA, Rodriguez JM, McDonald JF (2002). Evidence for the adaptive significance of an LTR retrotransposon sequence in a Drosophila heterochromatic gene. BMC Evol Biol.

[pbio-0060251-b035] Petrov DA, Aminetzach YT, Davis JC, Bensasson D, Hirsh AE (2003). Size matters: non-LTR retrotransposable elements and ectopic recombination in Drosophila. Mol Biol Evol.

[pbio-0060251-b036] Franchini LF, Ganko EW, McDonald JF (2004). Retrotransposon-gene associations are widespread among D. melanogaster populations. Mol Biol Evol.

[pbio-0060251-b037] Marsano RM, Caizzi R, Moschetti R, Junakovic N (2005). Evidence for a functional interaction between the Bari1 transposable element and the cytochrome P450 cyp12a4 gene in Drosophila melanogaster. Gene.

[pbio-0060251-b038] Aminetzach YT, Macpherson JM, Petrov DA (2005). Pesticide resistance via transposition-mediated adaptive gene truncation in Drosophila. Science.

[pbio-0060251-b039] Ganko EW, Greene CS, Lewis JA, Bhattacharjee V, McDonald JF (2006). LTR retrotransposon-gene associations in Drosophila melanogaster. J Mol Evol.

[pbio-0060251-b040] Ashburner M, Bergman CM (2005). Drosophila melanogaster: a case study of a model genomic sequence and its consequences. Genome Res.

[pbio-0060251-b041] Quesneville H, Bergman CM, Andrieu O, Autard D, Nouaud D (2005). Combined evidence annotation of transposable elements in genome sequences. PLoS Comput Biol.

[pbio-0060251-b042] David JR, Capy P (1988). Genetic variation of Drosophila melanogaster natural populations. Trends Genet.

[pbio-0060251-b043] Lachaise D, Cariou M-L, David JR, Lemeunier F, Tsacas F (1988). Historical biogeography of the Drosophila melanogaster species subgroup. Evol Biol.

[pbio-0060251-b044] Li H, Stephan W (2006). Inferring the demographic history and rate of adaptive substitution in Drosophila. PLoS Genet.

[pbio-0060251-b045] Thornton K, Andolfatto P (2006). Approximate Bayesian inference reveals evidence for a recent, severe bottleneck in a Netherlands population of Drosophila melanogaster. Genetics.

[pbio-0060251-b046] Orengo DJ, Aguade M (2004). Detecting the footprint of positive selection in a european population of Drosophila melanogaster: multilocus pattern of variation and distance to coding regions. Genetics.

[pbio-0060251-b047] Przeworski M (2002). The signature of positive selection at randomly chosen loci. Genetics.

[pbio-0060251-b048] Teshima KM, Coop G, Przeworski M (2006). How reliable are empirical genomic scans for selective sweeps. Genome Res.

[pbio-0060251-b049] Thornton KR, Jensen JD, Becquet C, Andolfatto P (2007). Progress and prospects in mapping recent selection in the genome. Heredity.

[pbio-0060251-b050] Macpherson JM, González J, Witten DM, Davis JC, Rosenberg NA (2008). Nonadaptive explanations for signatures of partial selective sweeps in Drosophila. Mol Biol Evol.

[pbio-0060251-b051] Kaminker JS, Bergman CM, Kronmiller B, Carlson J, Svirskas R (2002). The transposable elements of the Drosophila melanogaster euchromatin: a genomics perspective. Genome Biol.

[pbio-0060251-b052] Montgomery E, Charlesworth B, Langley CH (1987). A test for the role of natural selection in the stabilization of transposable element copy number in a population of Drosophila melanogaster. Genet Res.

[pbio-0060251-b053] Charlesworth B, Lapid A, Canada D (1992). The distribution of transposable elements within and between chromosomes in a population of Drosophila melanogaster. II. Inferences on the nature of selection against elements. Genet Res.

[pbio-0060251-b054] Sniegowski PD, Charlesworth B (1994). Transposable element numbers in cosmopolitan inversions from a natural population of Drosophila melanogaster. Genetics.

[pbio-0060251-b055] Hill WG, Robertson A (1966). The effect of linkage on limits to artificial selection. Genet Res.

[pbio-0060251-b056] Smith JM, Haigh J (1974). The hitch-hiking effect of a favourable gene. Genet Res.

[pbio-0060251-b057] Charlesworth B, Morgan MT, Charlesworth D (1993). The effect of deleterious mutations on neutral molecular variation. Genetics.

[pbio-0060251-b058] Hudson RR, Kaplan NL (1995). Deleterious background selection with recombination. Genetics.

[pbio-0060251-b059] Pool JE, Aquadro CF (2006). History and structure of sub-Saharan populations of Drosophila melanogaster. Genetics.

[pbio-0060251-b060] Andolfatto P, Przeworski M (2001). Regions of lower crossing over harbor more rare variants in African populations of Drosophila melanogaster. Genetics.

[pbio-0060251-b061] Sabeti PC, Reich DE, Higgins JM, Levine HZ, Richter DJ (2002). Detecting recent positive selection in the human genome from haplotype structure. Nature.

[pbio-0060251-b062] Innan H, Kim Y (2008). Detecting local adaptation using the joint sampling of polymorphism data in the parental and derived populations. Genetics.

[pbio-0060251-b063] Slatkin M, Rannala B (2000). Estimating allele age. Annu Rev Genomics Hum Genet.

[pbio-0060251-b064] Kidwell MG (1992). Horizontal transfer of P elements and other short inverted repeat transposons. Genetica.

[pbio-0060251-b065] Hoffmann AA, Weeks AR (2007). Climatic selection on genes and traits after a 100 year-old invasion: a critical look at the temperate-tropical clines in Drosophila melanogaster from eastern Australia. Genetica.

[pbio-0060251-b066] Papatsenko DA, Makeev VJ, Lifanov AP, Regnier M, Nazina AG (2002). Extraction of functional binding sites from unique regulatory regions: the Drosophila early developmental enhancers. Genome Res.

[pbio-0060251-b067] Al-Shahrour F, Minguez P, Tarraga J, Montaner D, Alloza E (2006). BABELOMICS: a systems biology perspective in the functional annotation of genome-scale experiments. Nucleic Acids Res.

[pbio-0060251-b068] Hubbard TJ, Aken BL, Beal K, Ballester B, Caccamo M (2007). Ensembl 2007. Nucleic Acids Res.

[pbio-0060251-b069] Wittkopp PJ, Haerum BK, Clark AG (2004). Evolutionary changes in cis and trans gene regulation. Nature.

[pbio-0060251-b070] Schaart JG, Mehli L, Schouten HJ (2005). Quantification of allele-specific expression of a gene encoding strawberry polygalacturonase-inhibiting protein (PGIP) using Pyrosequencing. Plant J.

[pbio-0060251-b071] Brizuela BJ, Elfring L, Ballard J, Tamkun JW, Kennison JA (1994). Genetic analysis of the brahma gene of Drosophila melanogaster and polytene chromosome subdivisions 72AB. Genetics.

[pbio-0060251-b072] Przeworski M, Coop G, Wall JD (2005). The signature of positive selection on standing genetic variation. Evolution.

[pbio-0060251-b073] Hermisson J, Pennings PS (2005). Soft sweeps: molecular population genetics of adaptation from standing genetic variation. Genetics.

[pbio-0060251-b074] Wray GA (2007). The evolutionary significance of cis-regulatory mutations. Nat Rev Genet.

[pbio-0060251-b075] Hoekstra HE, Coyne JA (2007). The locus of evolution: evo devo and the genetics of adaptation. Evolution.

[pbio-0060251-b076] Wittkopp PJ, Haerum BK, Clark AG (2008). Regulatory changes underlying expression differences within and between Drosophila species. Nat Genet.

[pbio-0060251-b077] Schlenke TA, Begun DJ (2004). Strong selective sweep associated with a transposon insertion in Drosophila simulans. Proc Natl Acad Sci U S A.

[pbio-0060251-b078] Clark AG, Eisen MB, Smith DR, Bergman CM, Oliver B (2007). Evolution of genes and genomes on the Drosophila phylogeny. Nature.

[pbio-0060251-b079] Begun DJ, Holloway AK, Stevens K, Hillier LW, Poh YP (2007). Population genomics: whole-genome analysis of polymorphism and divergence in Drosophila simulans. PLoS Biol.

[pbio-0060251-b080] Vogel C, Teichmann SA, Chothia C (2003). The immunoglobulin superfamily in Drosophila melanogaster and Caenorhabditis elegans and the evolution of complexity. Development.

[pbio-0060251-b081] Bustamante CD, Fledel-Alon A, Williamson S, Nielsen R, Hubisz MT (2005). Natural selection on protein-coding genes in the human genome. Nature.

[pbio-0060251-b082] Nielsen R, Hellmann I, Hubisz M, Bustamante C, Clark AG (2007). Recent and ongoing selection in the human genome. Nat Rev Genet.

[pbio-0060251-b083] Khlebodarova TM, Gruntenko NE, Grenback LG, Sukhanova MZ, Mazurov MM (1996). A comparative analysis of juvenile hormone metabolyzing enzymes in two species of Drosophila during development. Insect Biochem Mol Biol.

[pbio-0060251-b084] Flatt T, Tu MP, Tatar M (2005). Hormonal pleiotropy and the juvenile hormone regulation of Drosophila development and life history. Bioessays.

[pbio-0060251-b085] Lai EC (2004). Notch signaling: control of cell communication and cell fate. Development.

[pbio-0060251-b086] Kent D, Bush EW, Hooper JE (2006). Roadkill attenuates Hedgehog responses through degradation of Cubitus interruptus. Development.

[pbio-0060251-b087] Jia J, Jiang J (2006). Decoding the Hedgehog signal in animal development. Cell Mol Life Sci.

[pbio-0060251-b088] Maynard Smith JaH J (1974). The hitch-hiking effect of a favorable gene. Genet Res.

[pbio-0060251-b089] Kaplan NL, Darden T, Hudson RR (1988). The coalescent process in models with selection. Genetics.

[pbio-0060251-b090] Kaplan NL, Hudson RR, Langley CH (1989). The “hitchhiking effect” revisited. Genetics.

[pbio-0060251-b091] Berry AJ, Ajioka JW, Kreitman M (1991). Lack of polymorphism on the Drosophila fourth chromosome resulting from selection. Genetics.

[pbio-0060251-b092] Petrov DA (2002). DNA loss and evolution of genome size in Drosophila. Genetica.

[pbio-0060251-b093] Kimura M, Ota T (1969). The average number of generations until extinction of an individual mutant gene in a finite population. Genetics.

[pbio-0060251-b094] Rozen S, Skaletsky H (2000). Primer3 on the WWW for general users and for biologist programmers. Methods Mol Biol.

[pbio-0060251-b095] Lexa M, Horak J, Brzobohaty B (2001). Virtual PCR. Bioinformatics.

[pbio-0060251-b096] Haring E, Hagemann S, Pinsker W (1995). Different evolutionary behaviour of P element subfamilies: M-type and O-type elements in Drosophila bifasciata and D. imaii. Gene.

[pbio-0060251-b097] Wesley CS, Eanes WF (1994). Isolation and analysis of the breakpoint sequences of chromosome inversion In(3L)Payne in Drosophila melanogaster. Proc Natl Acad Sci U S A.

[pbio-0060251-b098] Kumar S, Tamura K, Nei M (2004). MEGA3: integrated software for Molecular Evolutionary Genetics Analysis and sequence alignment. Brief Bioinform.

[pbio-0060251-b099] Ewens WJ (2004). Mathematical population genetics: theoretical introduction.

[pbio-0060251-b100] Kreitman M (1983). Nucleotide polymorphism at the alcohol dehydrogenase locus of Drosophila melanogaster. Nature.

[pbio-0060251-b101] Schug MD, Hutter CM, Wetterstrand KA, Gaudette MS, Mackay TF (1998). The mutation rates of di-, tri- and tetranucleotide repeats in Drosophila melanogaster. Mol Biol Evol.

[pbio-0060251-b102] Tajima F (1983). Evolutionary relationship of DNA sequences in finite populations. Genetics.

[pbio-0060251-b103] Hudson RR (2002). Generating samples under a Wright-Fisher neutral model of genetic variation. Bioinformatics.

[pbio-0060251-b104] Tang H, Siegmund DO, Shen P, Oefner PJ, Feldman MW (2002). Frequentist estimation of coalescence times from nucleotide sequence data using a tree-based partition. Genetics.

[pbio-0060251-b105] Wang H, Elbein SC (2007). Detection of allelic imbalance in gene expression using pyrosequencing. Methods Mol Biol.

[pbio-0060251-b106] Peter A, Schottler P, Werner M, Beinert N, Dowe G (2002). Mapping and identification of essential gene functions on the X chromosome of Drosophila. EMBO Rep.

[pbio-0060251-b107] Schultz J, Copley RR, Doerks T, Ponting CP, Bork P (2000). SMART: a web-based tool for the study of genetically mobile domains. Nucleic Acids Res.

[pbio-0060251-b108] Augustin H, Grosjean Y, Chen K, Sheng Q, Featherstone DE (2007). Nonvesicular release of glutamate by glial xCT transporters suppresses glutamate receptor clustering in vivo. J Neurosci.

[pbio-0060251-b109] Gonzalez-Aguero M, Zuniga A, Pottstock H, Del Pozo T, Gonzalez M (2005). Identification of genes expressed during Drosophila melanogaster gastrulation by using subtractive hybridization. Gene.

[pbio-0060251-b110] Kamdar KP, Bachmann J, Broadus J, Stam L (2002). Nucleic acid sequences from Drosophila melanogaster that encode proteins essential for larval viability and uses thereof. World Patent 02057455.

