# Supplementary material for: High Rate of Recent Transposable Element–Induced Adaptation in Drosophila melanogaster
Source: PLoS Biol. 2008 Oct 21;6(10):e251. doi: 10.1371/journal.pbio.0060251 (PMC2570423; doi:10.1371/journal.pbio.0060251)
Supplement: Supplementary file 1 [file pbio.0060251.st003.pdf]

Table S3. Additional neutrality tests for each of the four TEs sequenced in this work. Within a cell, the upper number is the observed value of the statistics. The middle number is the mean, with the 2.5% and 97.5% confidence interval limits in parentheses and the lower number is the standard deviation.

	π_{NA}	π_{AF}	$\pi_{NA\text{-}TE}$	$\pi_{NA\text{-}non\ TE}$
FBti0018880	24.24<br>29.9 (7.85, 57.9)<br>12.8	103.2<br>100 (84.8, 110)<br>7.66	8.7<br>23.8 (3.62, 52.9)<br>12.8	63.19<br>28.6(3.16, 69.5)<br>18
FBti0019065	36.21<br>52.2 (41.3, 62.3)<br>5.39	NA	9.04<br>47.8 (33.6, 60)<br>6.83	44.67<br>48.9 (34.6, 61.6)<br>6.77
FBti0019170	41.72<br>73.7 (60.4, 86.5)<br>6.82	NA	24.3<br>67.4 (49.2, 83.3)<br>8.73	57.87<br>68.9 (48.9, 84.3)<br>9.02
FBti0019627	4.05<br>12.4 (2.64, 24.3)<br>5.56	40.09<br>46.4 (39.6, 53)<br>3.48	4.05<br>12.4 (2.64, 24.3)<br>5.56	NA
