# Supplementary material for: High Rate of Recent Transposable Element–Induced Adaptation in Drosophila melanogaster
Source: PLoS Biol. 2008 Oct 21;6(10):e251. doi: 10.1371/journal.pbio.0060251 (PMC2570423; doi:10.1371/journal.pbio.0060251)
Supplement: Supplementary file 2 [file pbio.0060251.st004.pdf]

Table S4. Population frequencies of the 13 putatively adaptive and eight putatively neutral TEs in two Australian populations. HP: number of strains homozygous for the presence of the element. HE: number of strains heterozygous. HA: number of strains homozygous for the absence of the element (see Materials and Methods). Significant p-values are highlighted in bold.

Flybase ID	Chromosomal location	Inversion	North population: Innisfail			Estimated frequency	South population: Yering Station			Estimated frequency	Total			Estimated frequency	ΔL	p-value
			HP	HE	HA		HP	HE	HA		HP	HE	HA			
FBti0019627	X(10C6-10C7)		6	13	3	0.57	7	9	2	0.67	13	22	5	0.60	0.4137	0.520
FBti0019056	X(12F1)		10	7	5	0.61	5	10	6	0.47	15	17	11	0.55	1.6427	0.200
FBti0019065	X(13C1)		2	5	14	0.21	6	8	7	0.47	8	13	21	0.35	6.4936	**0.011**
FBti0019170	2L(34C4)		0	8	14	0.18	2	15	4	0.44	2	23	18	0.31	7.4560	**0.006**
FBti0018880	2R(55F8)	In(2R)NS	1	17	2	0.48	6	15	1	0.65	7	32	3	0.55	1.6297	0.202
FBti0020046	3L(65A3)	In(3L)P	0	11	8	0.29	4	5	11	0.31	4	16	19	0.31	0.1156	0.734
FBti0020091	3L(68C1)	In(3L)P	6	13	1	0.63	13	7	0	0.87	19	20	1	0.73	4.0838	**0.043**
FBti0020119	3L(71E1)	In(3L)P	1	9	9	0.29	7	4	5	0.57	8	13	14	0.41	5.3855	**0.020**
FBti0019372	3R(88A4)		0	7	15	0.16	0	17	4	0.37	0	24	19	0.28	6.5873	**0.010**
FBti0019386	3R(89B7)		0	10	12	0.23	2	15	5	0.41	2	25	17	0.33	4.2180	**0.040**
FBti0019415	3R(94A3)	In(3R)P	5	7	7	0.45	12	4	2	0.80	17	11	9	0.61	8.7027	**0.003**
FBti0019430	3R(96D1)		11	11	0	0.75	13	9	0	0.84	24	20	0	0.77	0.2592	0.611
FBti0019443	3R(98B3)		0	5	17	0.11	1	18	3	0.44	1	23	20	0.28	13.2432	**0.000**
FBti0019164	2L(34A5)	In(2L)t	0	15	7	0.34	3	13	6	0.43	3	28	13	0.39	0.77	0.381
FBti0019624	X(10B2)		5	14	3	0.55	2	15	5	0.43	7	29	8	0.49	1.14	0.286
FBti0018879	2R(58E2)		7	13	2	0.61	6	12	0	0.67	13	25	2	0.64	0.24	0.623
FBti0019133	2L(28B2)	In(2L)t	4	14	4	0.50	2	19	0	0.55	6	33	4	0.52	0.20	0.658
FBti0019165	2L(34B1)		3	12	7	0.41	7	8	6	0.52	10	20	13	0.47	1.14	0.286
FBti0019410	3R(92E14)	In(3R)P	0	8	13	0.19	3	13	5	0.45	3	21	18	0.32	6.75	**0.009**
FBti0019604	X(7E4)		3	13	6	0.43	2	14	5	0.43	5	27	11	0.43	0.00	0.976
FBti0020057	3L(65E4)	In(3L)P	2	7	10	0.29	2	9	5	0.41	4	16	15	0.34	1.05	0.305
