# Supplementary material for: High Rate of Recent Transposable Element–Induced Adaptation in Drosophila melanogaster
Source: PLoS Biol. 2008 Oct 21;6(10):e251. doi: 10.1371/journal.pbio.0060251 (PMC2570423; doi:10.1371/journal.pbio.0060251)
Supplement: Supplementary file 3 [file pbio.0060251.st008.pdf]

Table S8. Primer pair combinations used to discover SNPs in the genes located close to 10 of the 13 adaptive TEs. For elements FBti0018880 and FBti0019627 SNPs previously discovered in this work were used.

Flybase ID	Name	Sequence (5'-3')	Name	Sequence (5'-3')
FBti0019170	kuz_L	GTGTTGCCCACAGTTGGTATA	kuz_R	CGCTGTTCCATTGTTGCAC
	kuz_L2	ACGATTACCCGCAGGAATGTC	kuz_R2	TCACGTGCGGCAGTGTGGTC
FBti0019065	CG18210_L	GTATCCACTTCTCGCATCGCT	CG18210_R	CGGATACGCAATTACGGAACA
	CG18210_L2	TTCGGGTTGAATTTGCATAAT	CG18210_R2	CCCGACTTTAGTTGCCATATT
FBti0019056	CG9413_L	GGGGATCACTACCACAGCTAT	CG9413_R	GCTGCCAAAGGTGCTGATG
FBti0019372	rdx_L	CCGGTGCTAATGACAAACTAA	rdx_R	GGTCTCTTACCTGCCAATATG
FBti0019386	CG6072_L	CGCCATATTCGATAAAAACG	CG6072_R	CCGCAATGGCATTGTCATAGT
FBti0020046	CG6467_L	GTGGCCGCCTCCGCTTTC	CG6467_R	GTAGGTTTGGGCGCACTTGGT
	CG6467_L2	GCATCACCGGAGGAAGCA	CG6467_R2	GGCCACGCAGAGGGTTGAGTC
FBti0020119	CG7439_L	CTCCTATTGAACGCGCTCCT	CG7439_R	GGGCCTGACCTTCCTCGATGC
	CG7439_L2	GGCAAGGCAGTCAACATAAGC	CG7439_R2	ACGCCATCTCGGTAGTAGA
FBti0019415	CG31163_L	CACACCACGCATACTCACAGC	CG31163_R	CAAGACCAAGACCCATGCC
FBti0019443	CG34353_L	CCTACAGTGCGCCGTAAAGTT	CG34353_R	GACTGCCGGCTGCGTGTTG
FBti0020091	CG6175_L	TTCCTTCCTTTGCCTTAAACC	CG6175_R	TGTTGCTGCTGCTCGAACTGC
	CG6175_L2	GATGTTGCCACGCTGCCACTC	CG6175_R2	GCGGTAAGCTCATCGTGTTGC
