# Supplementary material for: High Rate of Recent Transposable Element–Induced Adaptation in Drosophila melanogaster
Source: PLoS Biol. 2008 Oct 21;6(10):e251. doi: 10.1371/journal.pbio.0060251 (PMC2570423; doi:10.1371/journal.pbio.0060251)
Supplement: Supplementary file 4 [file pbio.0060251.st006.doc]

Table S6. *D. melanogaster* strains used in this study.

| Name of the pool | Strains in the pool | Geographical origin | Reference |
| --- | --- | --- | --- |
| Wi | wi1, wi3, wi15, wi18,wi41, wi45, wi68,wi77,wi83,wi98,wi137, wi148 | Wolfskill Orchard, Davis, CA, USA | Sergey Nuzhdin, |
| We1 | we4, we7, we10, we11, we25, we44, we47, we50, we57, we60, we67, we80 | Raleigh, NC, USA | Greg Gibson |
| We2 | we13, we17, we21 we28, we33, we37, we63, we70, we75, we83, we88, we91 | Raleigh, NC, USA | Greg Gibson |
| NA | Broward13, Broward5, Lake5, Okee14, Okee5, Orange1, Orange2, Paho4, Paho6, Paho9, Sebring12, Sebring17 | USA | Jeff Birdsley |
| NB | NB1, NB6, NB7, NB8, NB12, NB13, NB14, NB16 | New Buffalo, MI, USA | Bettina Harr |
| CSW | 3B, 6D, 11D, 20C, 23D, 25C, 29B, 36D | Countryside Winery, Blountville, TN, USA | Lev Yampolsky |
| MW | MW7, MW8, MW11, MW12, MW14, MW15, MW27, MW28, MW35, MW56, MW60 | Malawi |  |
| Zmel | zmel58, zmel81, zmel125,zmel131, zmel145, zmel159, zmel178, zmel191, zmel196 | Zimbabwe | Charles Aquadro |
| ZW | ZW104, ZW109, ZW122, ZW140, ZW141, ZW142, ZW144, ZW149, ZW155, ZW156, ZW177, ZW183 | Zimbabwe | Peter Andolfatto |
| KY | KY01, KY10, KY12, KY16, KY20, KY23, KY24, KY38, KY42, KY91, KY106 | Kenya | Peter Andolfatto |
| Innisfail | N3, N5, N16, N19, N26, N29, N30, N32, N38, N41, N42, N44, N46, N47, N48, N49, N50, N51, N52, N55, N61, N63 | Queensland, Australia | Ary A Hoffmann |
| Yering Station | S27, S30, S31, S41, S44, S47, S48, S95, S116, S131, S149, S154, S158, S191, S193, S197, S213, S216, S222, S224, S225, S313 | Victoria, Australia | Ary A Hoffmann |
