# Supplementary material for: High Rate of Recent Transposable Element–Induced Adaptation in Drosophila melanogaster
Source: PLoS Biol. 2008 Oct 21;6(10):e251. doi: 10.1371/journal.pbio.0060251 (PMC2570423; doi:10.1371/journal.pbio.0060251)
Supplement: Supplementary file 5 [file pbio.0060251.st007.doc]

Table S7. Primer pair combinations used to sequence the regions immediately 5’ and 3’ to the insertions sequenced in this work. The region amplified by primers 19627_L3 and R3 include the TE. Primer pair combinations used to sequence ~10 kb away regions 5’ and 3’ to the insertions are also given. The name of these primers contain an A letter.

| Name | Sequence (5’-3’) | Name | Sequence (5’-3’) |
| --- | --- | --- | --- |
| FBti0018880_L1 | CTTCATCCAAGGCGGAGATTG | FBti0018880_R1 | AGGTAGAAACGAGCCGCTGTC |
| FBti0018880_L2 | GACTTTGGCGCCATAGTCAC | FBti0018880_R2 | GTTGACCTTTTGTCGCCTTGC |
| FBti0018880_FL | CCTACACGGCGAGAAGAGAAAAT | FBti0018880_R | TTGTTGGCTTGTGGATTTCAAGT |
| FBti0018880_FL | CCTACACGGCGAGAAGAGAAAAT | FBti0018880_FLR | TTCACCTCCCCCCACTTGCT |
| FBti0018880_L | AGGGAGCCATCATTGTAATAGCG | FBti0018880_R | TTGTTGGCTTGTGGATTTCAAGT |
| FBti0018880_L3 | TCATTTTAAGCATGAATCGTT A | FBti0018880_R3 | TGATGTCGAAGGGTAGGA |
| FBti0018880_L4 | TGGGCAGATATGTATCGC | FBti0018880_R4 | CGCCGATCTGTGTATATAGA |
| FBti0019065_L1 | ATAAAGTGCGGCATTGTCTAT | FBti0019065_R1 | TTCGGCACTAGATCGGGTTGT |
| FBti0019065_L2 | TTGCCACTCATCAGATAATAG | FBti0019065_ R2 | CCAATCAATTTTGTTTAAGC |
| FBti0019065_FL | [CGATTCCATTCAATTCCATCTCA](http://www.fruitfly.org/cgi-bin/blast/public_blaster.pl?program=blastn&database=whole_genome_scaffolds_dmel_RELEASE3-1.FASTA&sequence=CGATTCCATTCAATTCCATCTCA&upfile=&title=&mail=BROWSER&email=&htmlform=TEXT&histogram=no&nscores=50&nalign=50&sort_by=pvalue&matrix=blosum62&expthr=default&cutoff=60&stats=sump&filter=All&strand=both&dbstrand=both) | FBti0019065_R | [TCGAAGTCTCCGTTGTTGTTGTT](http://www.fruitfly.org/cgi-bin/blast/public_blaster.pl?program=blastn&database=whole_genome_scaffolds_dmel_RELEASE3-1.FASTA&sequence=TCGAAGTCTCCGTTGTTGTTGTT&upfile=&title=&mail=BROWSER&email=&htmlform=TEXT&histogram=no&nscores=50&nalign=50&sort_by=pvalue&matrix=blosum62&expthr=default&cutoff=60&stats=sump&filter=All&strand=both&dbstrand=both) |
| FBti0019065_FL | [CGATTCCATTCAATTCCATCTCA](http://www.fruitfly.org/cgi-bin/blast/public_blaster.pl?program=blastn&database=whole_genome_scaffolds_dmel_RELEASE3-1.FASTA&sequence=CGATTCCATTCAATTCCATCTCA&upfile=&title=&mail=BROWSER&email=&htmlform=TEXT&histogram=no&nscores=50&nalign=50&sort_by=pvalue&matrix=blosum62&expthr=default&cutoff=60&stats=sump&filter=All&strand=both&dbstrand=both) | FBti0019065_FLR | GTCCAACGACACGCTTTGT |
| FBti0019065_L | [GCGCTCTTTAGTTCAAGATGCAA](http://www.fruitfly.org/cgi-bin/blast/public_blaster.pl?program=blastn&database=whole_genome_scaffolds_dmel_RELEASE3-1.FASTA&sequence=GCGCTCTTTAGTTCAAGATGCAA&upfile=&title=&mail=BROWSER&email=&htmlform=TEXT&histogram=no&nscores=50&nalign=50&sort_by=pvalue&matrix=blosum62&expthr=default&cutoff=60&stats=sump&filter=All&strand=both&dbstrand=both) | FBti0019065_R | [TCGAAGTCTCCGTTGTTGTTGTT](http://www.fruitfly.org/cgi-bin/blast/public_blaster.pl?program=blastn&database=whole_genome_scaffolds_dmel_RELEASE3-1.FASTA&sequence=TCGAAGTCTCCGTTGTTGTTGTT&upfile=&title=&mail=BROWSER&email=&htmlform=TEXT&histogram=no&nscores=50&nalign=50&sort_by=pvalue&matrix=blosum62&expthr=default&cutoff=60&stats=sump&filter=All&strand=both&dbstrand=both) |
| FBti0019065_L3 | AGCTGCATTTTTAAGTTACCT | FBti0019065_R3 | GCTCGAAGTCTCCGTTGTT |
| FBti0019065_L4 | GTTGTGTTTGCAACTAAATTG | FBti0019065_R4 | TGGTTGTTCGGTGACTC |
| FBti0019065_L5 | TCAAACGAGCGCAACCATACA | FBti0019065_R5 | GACAACGAGGGCGACAC |
| FBti0019170_L1 | AGGTTGCATCGCTGTTGAC | FBti0019170_R1 | ACAAAAAGCCATTCCATTGAC |
| FBti0019170_L2 | GCCCAGCTGATTGTCAT | FBti0019170_R2 | AAGAGAAATTGCAACGAAACA |
| FBti0019170_FL | GACGAATTCATAAATTGGCGGTT | FBti0019170_R | [TTGAATATTGTGTCGATTGCGTG](http://www.fruitfly.org/cgi-bin/blast/public_blaster.pl?program=blastn&database=whole_genome_scaffolds_dmel_RELEASE3-1.FASTA&sequence=TTGAATATTGTGTCGATTGCGTG&upfile=&title=&mail=BROWSER&email=&htmlform=TEXT&histogram=no&nscores=50&nalign=50&sort_by=pvalue&matrix=blosum62&expthr=default&cutoff=60&stats=sump&filter=All&strand=both&dbstrand=both) |
| FBti0019170_FL | GACGAATTCATAAATTGGCGGTT | FBti0019170_FLR | GAGAGTCGGCGTTCGTCATGC |
| FBti0019170_L | [TTCGGAGTGAAAACATCCAAAGA](http://www.fruitfly.org/cgi-bin/blast/public_blaster.pl?program=blastn&database=whole_genome_scaffolds_dmel_RELEASE3-1.FASTA&sequence=TTCGGAGTGAAAACATCCAAAGA&upfile=&title=&mail=BROWSER&email=&htmlform=TEXT&histogram=no&nscores=50&nalign=50&sort_by=pvalue&matrix=blosum62&expthr=default&cutoff=60&stats=sump&filter=All&strand=both&dbstrand=both) | FBti0019170_R | [TTGAATATTGTGTCGATTGCGTG](http://www.fruitfly.org/cgi-bin/blast/public_blaster.pl?program=blastn&database=whole_genome_scaffolds_dmel_RELEASE3-1.FASTA&sequence=TTGAATATTGTGTCGATTGCGTG&upfile=&title=&mail=BROWSER&email=&htmlform=TEXT&histogram=no&nscores=50&nalign=50&sort_by=pvalue&matrix=blosum62&expthr=default&cutoff=60&stats=sump&filter=All&strand=both&dbstrand=both) |
| FBti0019170_L3 | CACCTGGTATTACAAGTCAGT | FBti0019170_R3 | CGGTGGTGCAATCAACT |
| FBti0019170_L4 | AGCTTTTGCTCTCCGTTTTT | FBti0019170_R4 | CTCCAGCGATTATTGTTATGC |
| FBti0019627_L1 | CGATCGCATAAATCATTATT | FBti0019627_R1 | GATCACCGCTCGATAGAT |
| FBti0019627_L2 | TCGTACGCCGCAGTG | FBti0019627_R2 | TGTTCCGCATGAATAGGTAG |
| FBti0019627_L3 | TCGTTGGCTTTCACAAAC | FBti0019627_R3 | ACCTAACGCCTGAGTAGACC |
| FBti0019627_L4 | CGAAAGAAATGAAAACCGAT | FBti0019627_R4 | CCAAGAAGACGGATAGCAA |
| FBti0019627_L5 | GCGCCCTCGTCCATCA | FBti0019627_R5 | AGGGTTGCCAGACTGACGTT |
| FBti0018880_A1 | CGGGTTGAAAGTGATGTC | FBti0018880_A2 | TTGGTTGCTTAGCAGTTAAAT |
| FBti0018880_A3 | TTTGGCTGCAGAATACTTATG | FBti0018880_A4 | CGCCTGACCTACGACACTA |
| FBti0019627_A1 | GCGGGCAACGAGGCTACATT | FBti0019627_A2 | TGTGGATCTGGCCGGTTCA |
| FBti0019627_A3 | GAGGGCGAGGAAAAACGTA | FBti0019627_A4 | TGCATCCAGTGGTTAGCGTA |
| FBti0019065_A1 | GGCGGCATTAATTGGGCTTA | FBti0019065_A2 | CGAGTTGGCCAAGTTTTCGT |
| FBti0019065_A3 | GGGTGGTGTTCGCATGT | FBti0019065_A4 | TCGGGTCGAGGGCTATAATA |
| FBti0019170_A1 | CGAAGAAGTACACAAGCTCA | FBti0019170_A2 | CCCGCTCGCACATTA |
| FBti0019170_A3 | TTAGTTGGAATCGGGTAG | FBti0019170_A4 | AAAGCATTCCAATCATTATT |
